# Enhancing the Printability of Laser Powder Bed Fusion-Processed Aluminum 7xxx Series Alloys Using Grain Refinement and Eutectic Solidification Strategies

**DOI:** 10.3390/ma18225089

**Published:** 2025-11-09

**Authors:** Chukwudalu Uchenna Uba, Huan Ding, Yehong Chen, Shengmin Guo, Jonathan Richard Raush

**Affiliations:** 1 Department of Mechanical Engineering, University of Louisiana at Lafayette, Lafayette, LA 70503, USA; jraush@louisiana.edu; 2 Department of Mechanical and Industrial Engineering, Louisiana State University, Baton Rouge, LA 70803, USA; hding3@lsu.edu (H.D.); yche136@lsu.edu (Y.C.); sguo2@lsu.edu (S.G.)

**Keywords:** Al 7075 alloy, hot cracking susceptibility, hot susceptibility index, cracking susceptibility coefficient, columnar-to-equiaxed transition, laser powder bed fusion, grain refinement, eutectic solidification, selective laser melting

## Abstract

As the most commercially developed metal additive process, laser powder bed fusion (LPBF) is vital to advancing several industry sectors, enabling high-precision part production across aerospace, biomedical, and manufacturing industries. Al 7075 alloy offers low density and high-specific strength yet faces LPBF challenges such as hot cracking and porosity due to rapid solidification, thermal gradients, and a wide freezing range. To address these challenges, this study proposes an integrated computational and experimental framework to enhance the LPBF processability of Al 7xxx alloys by compositional modification. Using the Calculation of Phase Diagram approach, printable Al 7xxx compositions were designed by adding grain refiners (V and/or Ti) and a eutectic solidification enhancer (Mg) to Al 7075 alloy to enable grain refinement and eutectic solidification. Subsequent LPBF experiments and characterization tests, such as metallography (scanning electron microscopy), energy-dispersive X-ray spectroscopy, X-ray diffraction, and X-ray micro-computed tomography, confirmed the production of refined microstructures with reduced defects. This study contributes to existing approaches for producing high-quality Al 7xxx alloy parts without significant compositional deviations using an integrated computational and experimental approach. Finally, aligning with the Materials Genome Initiative, this study contributes to the development and industrial adoption of advanced materials.

## 1. Introduction

Metal additive manufacturing (AM) has evolved rapidly since its emergence in the late 1980s and early 1990s [1], becoming increasingly important across medical, aerospace, defense, automotive, oil and gas, and tooling industries. This widespread adoption is driven by its ability to produce topologically complex net or near-net shapes with design freedom [2,3,4,5,6], reduced lead time, and decreased manufacturing costs compared to conventional approaches [7,8]. The layer-wise processing approach and rapid solidification in AM produce distinct microstructures that can, in some cases, yield improved mechanical [9,10,11,12] and corrosion resistance [13,14] properties. Selective laser melting (SLM), an LPBF technology, can fabricate fully dense metallic parts from powders [15] and is particularly suited for alloys that are difficult to shape conventionally, including aluminum [16], stainless steel [17], titanium [18,19], cobalt–chrome [20], and nickel [21] alloys. Despite these advancements, Al alloy-based structural parts are mainly manufactured by casting, forging, extrusion, and powder metallurgy [22]. However, increasing demands for lightweight, high-performance designs have intensified research interests in AM [23,24]. Thus, producing Al alloy parts with diverse structures, high dimensional accuracy, and near-net-shape using AM remains a major research focus.

During LPBF, the rapid solidification of metal alloys leads to unique structural and mechanical properties with high heating and cooling rates (10^3^–10^8^ K·s^−1^) [25,26]. This rapid processing results in constitutional changes, such as partitionless solidification and microstructural individual phase refinements, dependent on the solidification interface velocity [27,28]. The LPBF processing of Al alloys has gained much interest in recent years, mainly due to the achievable peculiar microstructures and enhanced mechanical properties [29]. However, only a few Al alloys have been processed successfully and crack-free by LPBF, such as the low-strength and low-ductility (∼4%) Al–Si7–Mg0.6, Al–Si9–Mg0.3, Al–Si10–Mg, and Al–Si12 alloys, which is due to their excellent castability, with low shrinkage due to the large fraction of Al–Si eutectic [30]. Concerning Al 7075 alloy, its LPBF processability is limited due to its low weldability, resulting from hot-cracking and stress corrosion due to the fast solidification rate [31]. During ultrafast solidification, the primary equilibrium phase solidifies at a different composition from the bulk liquid, resulting in solute enrichment in the liquid near the solidifying interface, locally changing the equilibrium liquidus temperature and producing an unstable, undercooled condition. Consequently, there is a breakdown of the solid–liquid interface, leading to cellular or dendritic grain growth with long channels of interdendritic liquid trapped between solidified regions. As temperature and liquid volume fraction decrease, volumetric solidification shrinkage and thermal contraction in these channels produce cavities and hot tearing cracks, which may span the entire length of the columnar grain and can propagate through additional intergranular regions [32].

Although processing parameter optimization can vary the solidification conditions during LPBF and improve part quality, it cannot eliminate cracking entirely due to the intrinsic high thermal gradient and large thermal stresses [33]. For example, in a holistic study, Stopyra et al. [34] conducted multistage trials to adjust the optimum process parameters to produce crack-free LPBF-processed Al 7075 parts with no success. Fortunately, cracking can be eliminated by refining the large columnar grains into homogeneous, fine equiaxed grains with higher cracking resistance by inoculation treatment [33,35]. This approach entails introducing lattice-matched nucleation particles and/or solutes with a high growth restriction factor into the original alloys to achieve columnar-to-equiaxed transition (CET) during LPBF [36,37,38]. LPBF-processed high-strength Al alloys have been developed by introducing alloying elements with high growth restriction factors (e.g., Sc, Zr, Er, Ti, V, Nb, and Ta), metal-based nanoparticles, or ceramic particles (e.g., TiC, TiN, TiCN, and TiB_2_) [33,38,39,40,41,42,43,44,45,46,47]. The eutectic solidification (ES) strategy is another approach that has been widely implemented in Al–Si [48,49,50] and Al–Ce [51,52] alloys. In this approach, ES is facilitated by introducing eutectic-forming solute elements in Al, which provides ample liquid for the backfilling of cracks and is specifically effective if it occurs in the terminal solidification stage (the stage with the highest hot cracking susceptibility (HCS)) [35]. Although studies have obtained promising results using these approaches, some utilized expensive metal-based nanoparticles (XH_1.5_ or XH_2_, where X represents the metal atom), which introduced gas holes that deteriorate densification behavior. Some utilized expensive elements (such as Sc [36,37], Zr, and Ta [43]) and ceramic nanoparticles (TiC/Ti [53], TiN/Ti [54], and TiB_2_/Si [55]). Note that some of these novel methods often require additional processes, such as ball milling for extended durations, before LPBF [47]. Some utilized large amounts of foreign nucleant particles or solute elements, such as 5–16 wt.% Si. [56,57], causing embrittlement and/or compositional deviation, thereby creating post-processing and application difficulties. To the best of our knowledge, only one study exists where the synergy of grain refinement and ES strategies has been applied to process crack-free LPBF-processed Al 7075 alloy. Li et al. [58] developed a novel SLM-processed Si-modified (2.9 wt.%) and Zr-modified (1 wt.%) Al–Zn–Mg–Cu (Al 7075) alloy. However, their study provided no computational thermodynamic basis for the selection and amount of the alloying elements; instead, it used trial-and-error experimental approaches, which are costly and time-consuming. Thus, the question begs whether it is possible to fabricate crack-free/crack-reduced LPBF-processed Al 7075 alloy by grain refinement and ES strategies using an integrated experimental and computational approach to save time and cost.

Therefore, a key research gap in the literature lies in the absence of a computationally guided, composition-level alloy design strategy that integrates heterogeneous nucleation (HN) and eutectic feeding for LPBF-processed Al 7xxx alloys, which eliminates trial-and-error experimental approaches, avoids excessive solute additions, and is practically effective. To bridge this gap, the study develops an integrated computational and experimental framework that couples grain refinement (by HN on potent primary particles, resulting in CET) and ES [35]. Grain refiners, such as V and Ti, and a eutectic-forming element, such as Mg, were added to Al 7075 alloy in trace amounts. V and Ti were selected as grain refiners because they facilitate the formation of L1_2_ primary trialuminides (Al_3_V and Al_3_Ti); the small degree of lattice parameter misfit between them and Al makes them potent nuclei for HN. Mg was selected as the eutectic-forming solute element in Al because it would solidify via ES over the large part of the final stage or throughout the final stage, thus likely exhibiting crack-free microstructure and a wider processing window [36]. In LPBF, the cooling rates are very high, but the ES occurs at a specific or narrow temperature range. Thus, the deleterious effects of thermal contraction strains are minimized. This study is based on the premise that the combined use of V/Ti and Mg will reduce solidification cracking and porosity by simultaneously promoting equiaxed grain formation and sufficient terminal liquid feeding. Specifically, it is proposed that during solidification (i) trace V and Ti additions generate adequate Al_3_V and Al_3_Ti nuclei to encourage CET under LPBF thermal gradients, (ii) Mg enrichment enables the timely formation of the Al_2_Mg_3_Zn_3_ eutectic fraction toward the end of solidification at a relatively higher temperature and lower solid fraction, allowing ES to persist to the end and improving liquid feeding to reduce defects, and (iii) a Calculation of Phase Diagram (CALPHAD)-guided alloy design framework can achieve these improvements more efficiently than process-parameter optimization achieved by trial-and-error experimental approaches. 

To accomplish this study, first, LPBF-processible Al 7xxx alloys were designed by introducing grain refiners and an ES enhancer to Al 7075 alloy using the CALPHAD approach. Second, cuboids of the compositions of interest were printed using LPBF. Finally, metallography (scanning electron microscopy (SEM)), energy-dispersive X-ray spectroscopy (EDS), X-ray micro-computed tomography (micro-CT), and X-ray diffraction (XRD) studies were conducted to characterize the morphologies of the as-printed samples, the elemental distribution, the sizes and distributions of the internal voids within the as-printed samples, and the phases in the as-printed samples, respectively. This study contributes to existing approaches for producing LPBF-processible Al 7xxx alloy parts without significant compositional deviations using an integrated computational and experimental approach. It distinctively presents a cost-effective, composition-level “nucleation-and-feeding” co-design strategy actualized using a CALPHAD–LPBF–characterization workflow that links predicted solidification metrics to observed cracking and porosity. Furthermore, it contributes to the Materials Genome Initiative by demonstrating a practical pathway for designing LPBF-processible high-strength aluminum alloys, thereby supporting the development and industrial adoption of advanced materials.

## 2. Materials and Methods

### 2.1. CALPHAD-Based Alloy Design Approach for LPBF

Herein, LPBF-processible Al 7xxx alloys were designed by conducting CALPHAD-based thermodynamic simulations using the Scheil solidification simulation and property modules of the Thermo-Calc 2024a software package and the corresponding TCAL8: Al-Alloys v8.2 Thermodynamic and MOBAL7: Al-Alloys Mobility v7.0 databases.

First, classic Scheil simulations were conducted to simulate the liquid-phase composition and solidification path (temperature–mole fraction of solid (*T*–$f_{S}$) and *T*–$f_{S}^{1/2}$ curves) during solidification. These curves present a cost-effective, high-throughput method for computationally assisted alloy design for AM [35]. The solidification path helps provide insights about HCS, and the amount of grain-refining or eutectic-forming phases can be predicted [35]. The classic Scheil model assumes infinitely fast solute diffusion in the liquid, no solute diffusion in the solid, and a liquid–solid interface in thermodynamic equilibrium. Consequently, classic Scheil calculations predict an extreme case of solute segregation during the solidification process. During the calculation process, negligible solid-state diffusion was assumed because of the high solidification rate of the LPBF process, while convective-driven Marangoni flows in the melt pool foster liquid mixing [35,59,60,61], which is favorable for applying the Scheil simulation. This assumption may not always be appropriate for conventional manufacturing processes, such as casting. However, when solidification rates match the solute atom diffusion speed across the solid–liquid interface, solute trapping may occur, making perfect liquid mixing challenging. Under such conditions, using a growth rate-dependent partition coefficient becomes essential [59]. Thus, the classic Scheil simulation-generated *T*–$f_{S}$ curves represent a limiting case of solidification in LPBF, with complete solute trapping being another limit [35]. The simulation spanned from the liquidus temperature to the solidus temperature. During the simulations, a temperature step of 1 °C was used, and the solidification was terminated when the liquid phase fraction was less than 0.01. The solid fraction was sequentially calculated per the temperature. Herein, several classic Scheil simulations were conducted while adding different amounts of Ti, V, and/or Mg to select the compositions of interest. 

Second, classic Scheil-based thermodynamic simulations were conducted to compute the cracking susceptibility coefficients (CSCs), freezing ranges, hot susceptibility index (HSI; Equation (1)), critical temperatures (Δ*T*_CTR_; Equation (2)), and liquidus and solidus temperatures as a function of the base alloy and different additions of alloying elements.

(1) $$\mathrm{HSI}=\left|\frac{dT}{df_{S}^{1/2}}\right|\;near\;f_{S}^{1/2\;}=1,$$

(2) $$∆T_{\mathrm{CTR}}=T_{f_{S}=0.95}\;-\;T_{f_{S}=1}.$$

Here, the freezing range is the difference between the liquidus and solidus temperatures on the *T*–$f_{S}$ curve, HSI is the absolute value of the slope of the *T*–$f_{S}^{1/2}$ curve near $f_{S}^{1/2}$ = 1 [62,63], and $∆T_{\mathrm{CTR}}$ is the difference between the temperatures corresponding to $f_{S}$ = 0.95 ($T_{f_{S}\;=\;0.95}$) and $f_{S}$ = 1 ($T_{f_{S}\;=\;1}$) [62]. HSI and $\text{∆}T_{\mathrm{CTR}}$ both focus on the terminal stage of solidification. A high HSI value represents poor bridging capability of grains and thus poor printability [64,65], and a high $∆T_{\mathrm{CTR}}$ value indicates that the alloy spends a longer time in the most cracking susceptible stage and that higher contraction strains exist [66]. The HSI calculation in Thermo-Calc is based on Kou’s criterion. Kou [65] considered (1) the separation of grains under tensile strain resulting in cracking, (2) the growth of grains, thereby bridging together to resist cracking, and (3) liquid feeding along the grain boundaries to resist cracking, and showed that $\left|\frac{dT}{df_{S}^{1/2}}\right|$ near $f_{S}^{1/2}$ = 1 of an alloy can be an index for its crack susceptibility. He concluded that a high $\left|\frac{dT}{df_{S}^{1/2}}\right|$ near $f_{S}^{1/2}$ = 1 discourages the bridging of neighboring columnar dendritic grains needed to resist cracking by slowing down the lateral growth rate of the grains. It also discourages the shrinkage feeding needed to resist cracking by lengthening the grain-boundary channel to hinder liquid flow through the channel. The CSC calculation is based on the Clyne and Davies model, which describes the effects of alloy composition on hot tearing, and is combined with a heat flow model proportional to 1/*t*^0.5^, where *t* represents time [67,68,69,70]. Solidification cracking happens in the later stage of solidification. With combined solidification and heat flow models, CSC is defined as follows:

(3) $$\mathrm{CSC}\;=\frac{t_{V}}{t_{R}},$$

where $t_{R}$ is the relaxed period when the material is less prone to cracking (liquid-phase amount = 60%–10%) due to adequate liquid feeding to the grain gaps caused by thermal contraction, and $t_{V}$ is the duration when the material is vulnerable to cracking due to limited availability of liquid (liquid-phase amount = 10%–1%) [67,68,69,70].

### 2.2. Compositions of Interest

Guided by CALPHAD-based thermodynamic simulations, grain refiners (V and Ti) and a eutectic enhancer (Mg) were added to Al 7075 alloy in trace amounts. Table 1 presents the compositions of interest. V, Ti, and Mg powders are cost-effective and commercially available; thus, their adoption represents a cost-effective approach to designing LPBF-processible Al 7xxx alloys. The total amount of alloying elements was set to ≤4 wt.% to avoid significant compositional deviation. Tan et al. [33] showed that the addition of 1 wt.% Ti to Al 7075 promoted in situ formation of L1_2_-Al_3_Ti, which refined the grains. Thus, 1 wt.% V and 1 wt.% Ti were adopted to ensure comparable nucleant densities, adequate dispersion within LPBF melt pools, and to assess the combined effect of Al_3_V and Al_3_Ti nucleants on grain refinement. Also, previous studies have shown that modest Mg additions (~1–2 wt.%) in Al 7075 improve melt stability, porosity reduction, and densification during AM [71,72]. Thus, 0–2 wt.% Mg was adopted to characterize the effect of increasing Mg content on the formation *T* and $f_{S}$ of the Al_2_Mg_3_Zn_3_ eutectic toward the end of solidification.

### 2.3. Materials

Feedstock materials included a high-strength Al 7075 alloy plate of dimensions 304.8 mm × 304.8 mm × 9.52 mm (McMaster-Carr, Atlanta, GA, USA); spherical Al 7075 alloy powder of particle sizes of 10–50 µm (American Elements, Los Angeles, CA, USA) (Table 2 shows the alloy’s elemental composition); titanium, magnesium, and vanadium powders of 99.8%, 99.5%, and 99.5% purity values and particle sizes of <44 µm, respectively (Fisher Scientific, part of Thermo Fisher Scientific Inc., Waltham, MA, USA); sodium fluoride powder and sodium hydroxide pellets (MilliporeSigma, Burlington, MA, USA); Weck’s reagent (Reagents: A Part of TCP Analytical, Belmont, NC, USA).

### 2.4. Sample Preparation

Pure spherical Al 7075 alloy, Ti, Mg, and V powder samples were measured in the correct proportions according to the compositions of interest (Table 1) using a Mettler Toledo XP105 analytical balance (Mettler Toledo, Columbus, OH, USA). Next, the samples were mixed for 10 h in an MSK-SFM-3 Desktop High-speed Vibrating Ball Mill Mixer (MTI Corporation, Richmond, CA, USA) to ensure the homogenous distribution of the alloying powder particles on the Al 7075 alloy powder surface. For the LPBF experiments, several square substrates of 20 mm × 20 mm × 8 mm were cut from the high-strength Al 7075 alloy plate using a MAXIEM 1515 Abrasive Waterjet equipment (OMAX, Kent, WA, USA).

### 2.5. LPBF Experiments

LPBF-processed cuboid specimens of 10.5 mm × 9 mm × 5 mm were fabricated from the uniformly mixed compositions of interest using a custom-built LPBF machine (Metal Additive Manufacturing Laboratory, Louisiana State University, Baton Rouge, LA, USA) under a flowing argon atmosphere. The custom-built machine, which was designed and assembled by Wen et al. [73,74], is equipped with a ytterbium fiber laser of 1070 nm wavelength and in the continuous mode (IPG model: YLR-200-AC-Y11; IPG Photonics, Marlborough, MA, USA), a D25 (25-mm diameter) Collimator (IPG Photonics, Marlborough, MA, USA), a ProSeries II scan head (Cambridge Technology Inc., Bedford, MA, USA), which is controlled by ScanMaster software (Version: A Draft 2014; Cambridge Technology Inc., Bedford, MA, USA), and a Jenoptik F-theta lens with flange focus length of 445.8 mm and minimum spot size of 46 μm (Jenoptik Optical Systems, LLC, Jupiter, FL, AL, USA). The detailed preparation, setup, and operational procedures for the machine have been described in Refs [73,74]. The samples were printed at a laser power *P* of 168 W, which is the maximum power of the equipment. Average literature-obtained process parameter values for processing Al 7075 alloys are laser power *P* of 330–350 W, scan speed *v* of 800–1400 mm·s^−1^, hatch spacing *h* of 0.05–0.17 mm, and layer thickness *t* of 0.03–0.05 mm [33,36,47,56,57,58,75,76,77,78]. Thus, hatch spacing *h* and layer thickness *t* values of 0.07 and 0.05 mm were used herein. Furthermore, a scan speed *v* of 500 mm·s^−1^ was used, which was obtained by relating the volumetric energy density (VED) of the average literature-obtained laser process parameters to the maximum laser power of the equipment. Then, the corresponding speed was calculated using Equation 4, where *P* is the laser power (W), *v* is the scanning speed (mm·s^−1^), *h* is the hatch spacing (mm), and *t* is the layer thickness. A simple ‘back-and-forth scanning strategy’ was used without rotation across layers.

(4) $$\mathrm{VED}=\frac{P}{v\;\times \;h\;\times \;t}$$

### 2.6. Microstructural Characterization

The cross-sectional surfaces (parallel to the build direction) of the cuboid specimens were ground using silicon carbide paper of grit sizes US 240, 320, 400, 600, and 1200, respectively, and were polished to a mirror finish using Microcloth PSA 12 inches (Diamond size: 5–0.02 μm), colloidal silica, and an Allied M-Prep Manual Grinder/Polisher Machine (Allied High Tech Products, Cerritos, CA, USA). Next, the samples were etched via a two-step method: first, a pre-etch step by immersing in caustic sodium fluoride (93 mL of deionized water + 2 g NaOH + 5 g NaF) for 5 min. Afterward, the samples were rinsed in deionized water and ethanol, respectively. Second, the coloring step was actualized by immersing the samples in Weck’s reagent for 30 s. Afterward, the samples were rinsed in deionized water and ethanol and left to dry at room temperature (22 °C).

The morphologies of the etched samples were investigated using SEM (Thermo Scientific Scios 2 Dual Beam SEM and Focused Ion Beam with Thermo Scientific UltraDry Energy-Dispersive Spectrometry (Pathfinder 2.8 X-ray Microanalysis software), Waltham, MA, USA). EDS (PathFinder X-ray Micro Analysis, Thermo Scientific Scios 2 Dual Beam SEM, and Focused Ion Beam with Thermo Scientific UltraDry Energy-Dispersive Spectrometry, Waltham, MA, USA) was used to obtain the spectra, quantitative elemental compositions, grayscale images, and the elemental mappings of the samples. Three-dimensional visualizations for determining the size and distribution of internal cracks/pores within as-printed specimens were conducted using a ThermoFisher HeliScan X-ray micro-CT system (Waltham, MA, USA). Cuboid samples of average dimensions of ~8 mm × 7 mm × 4 mm of the compositions of interest were examined using X-ray micro-CT. The generated data were reconstructed, and the Z-stacked generated images were analyzed using Avizo 2022.2 software. Finally, XRD examinations were performed on a PANalytical Empyrean system (Boulder, CO, USA) with Cu Kα radiation to analyze the phases of the compositions of interest. Symmetric *θ*/2*θ* scans were conducted in the angular range of 20°–120° 2*θ*, using a continuous scanning strategy and a scanning step size of 0.013°.

## 3. Results and Discussion

### 3.1. CALPHAD-Based Alloy Design Analysis for LPBF Using Solidification Indices

**Figure 1:**
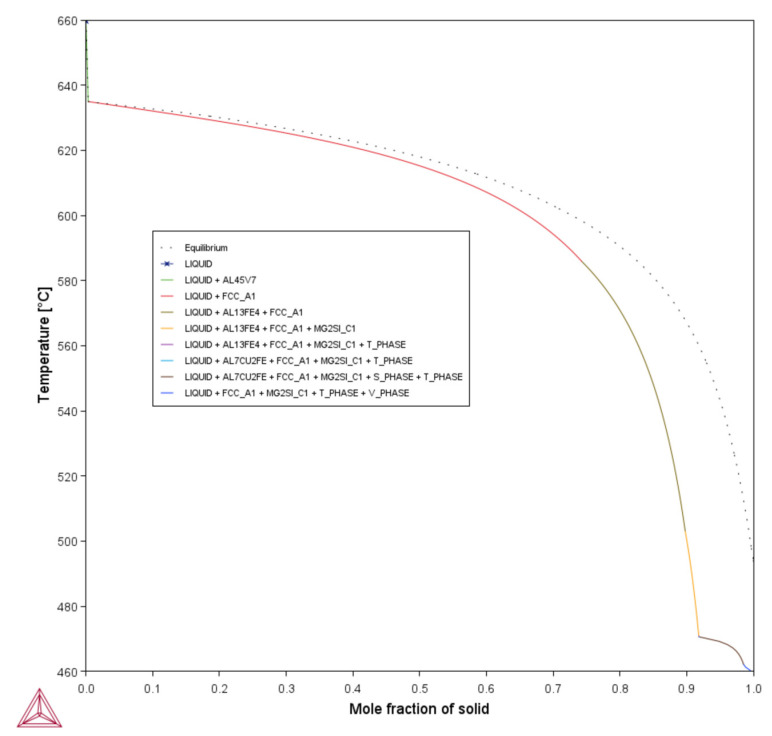
T–*f*_S_ curve of Composition 1 (Al 7075 alloy).

**Figure 2:**
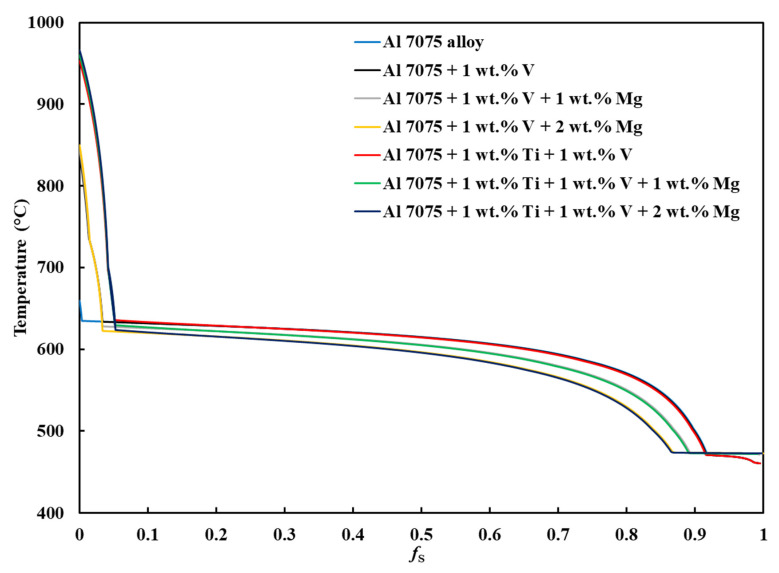
T–*f*_S_ curves of Al 7xxx alloys from *f*_S_ = 0 to *f*_S_ = 1.

**Figure 3:**
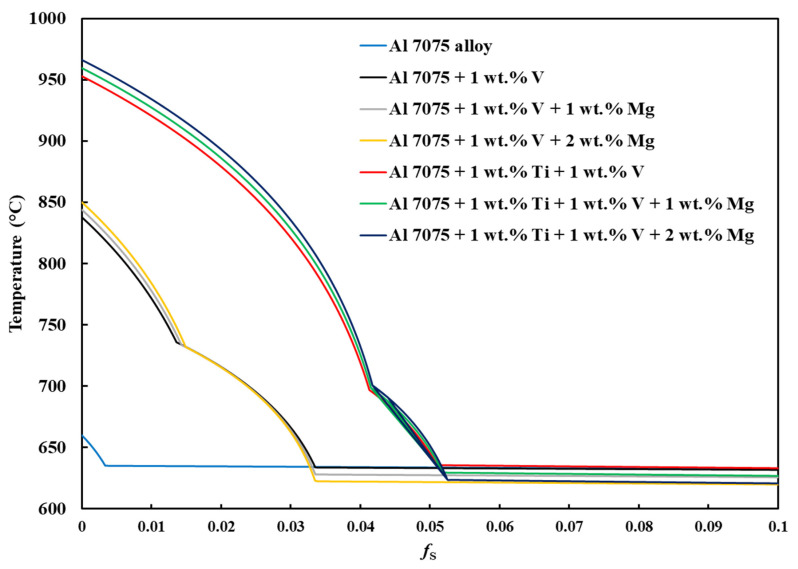
*T*–*f*_S_ curves of Al 7xxx alloys from *f*_S_ = 0 to *f*_S_ = 0.1.

**Figure 4:**
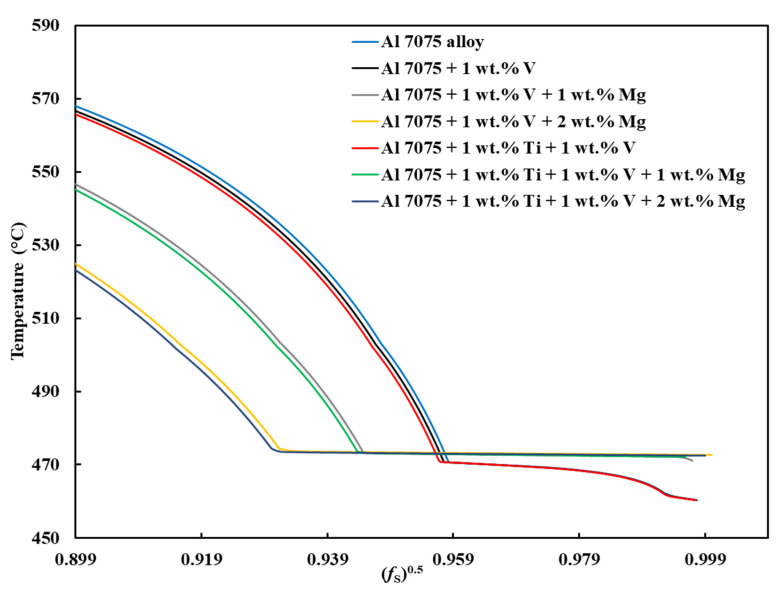
T–$f_{S}^{0.5}$ curves of Al 7xxx alloys near $f_{S}^{0.5}$ = 1.

**Figure A1:**
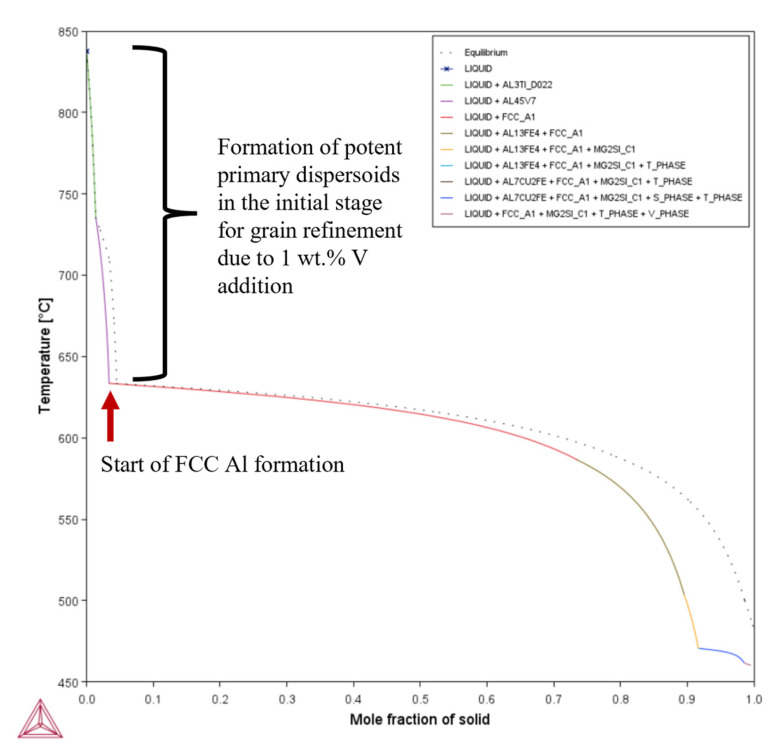
*T*–*f*_S_ curve of Composition 2 (Al 7075 + 1 wt.% V alloy).

**Figure A2:**
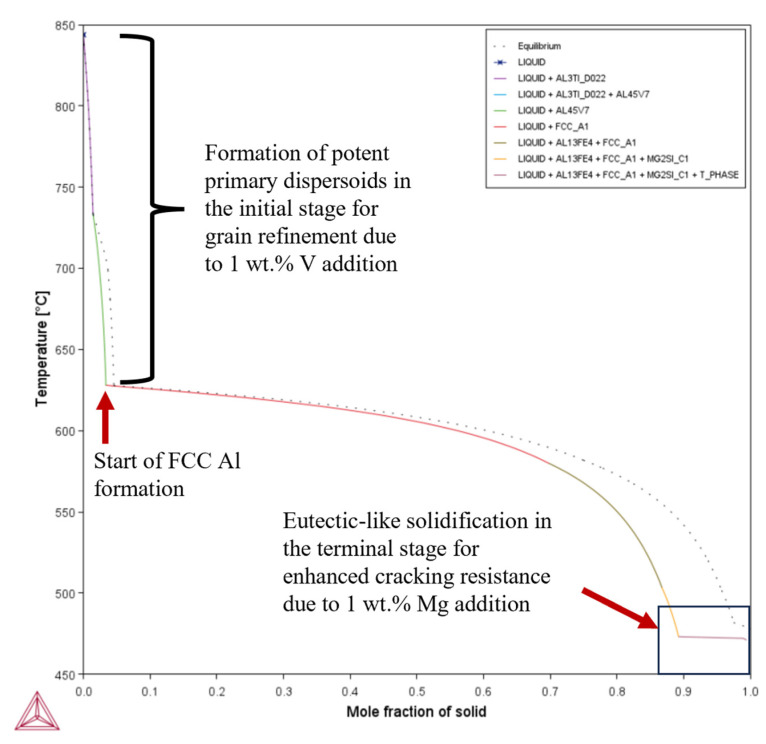
*T*–*f*_S_ curve of Composition 3 (Al 7075 + 1 wt.% V + 1 wt.% Mg alloy).

**Figure A3:**
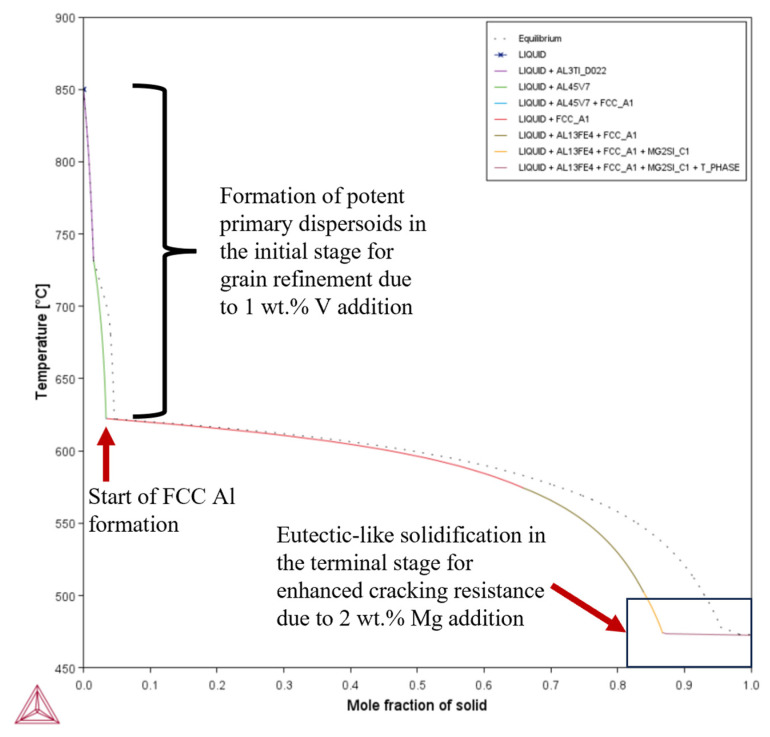
*T*–*f*_S_ curve of Composition 4 (Al 7075 + 1 wt.% V + 2 wt.% Mg alloy).

**Figure A4:**
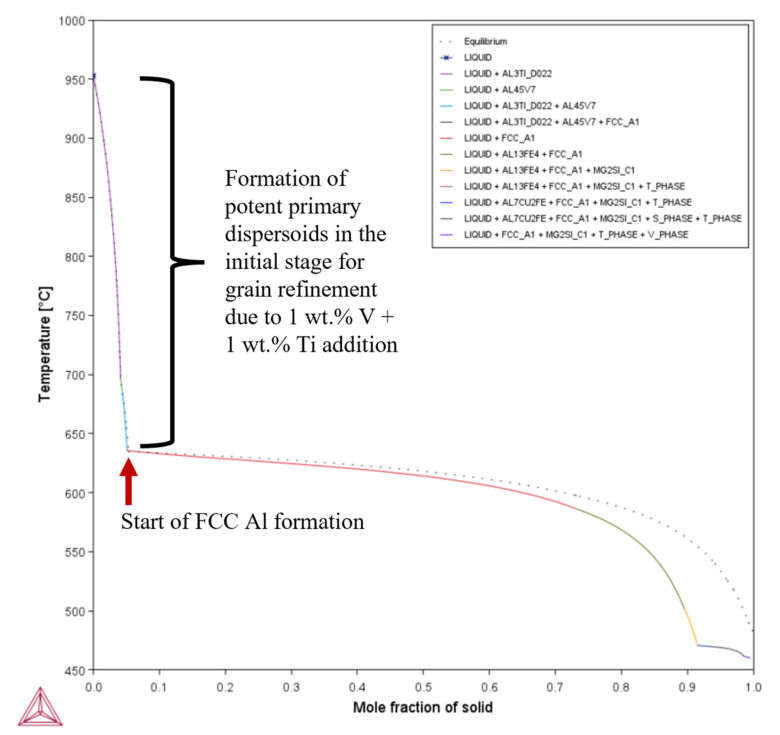
*T*–*f*_S_ curve of Composition 5 (Al 7075 + 1 wt.% V + 1 wt.% Ti alloy).

**Figure A5:**
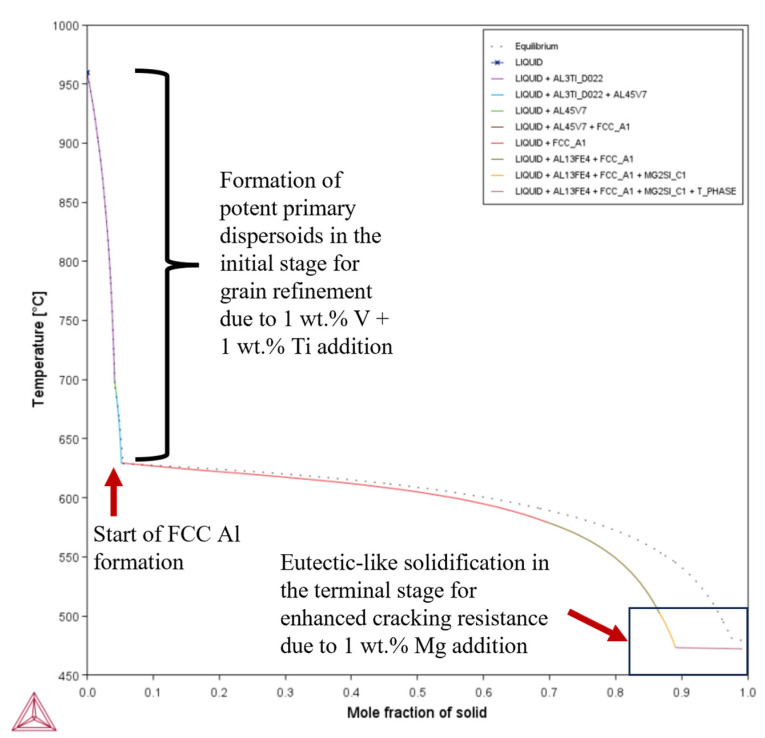
*T*–*f*_S_ curve of Composition 6 (Al 7075 + 1 wt.% V + 1 wt.% Ti. + 1 wt.% Mg alloy).

**Figure A6:**
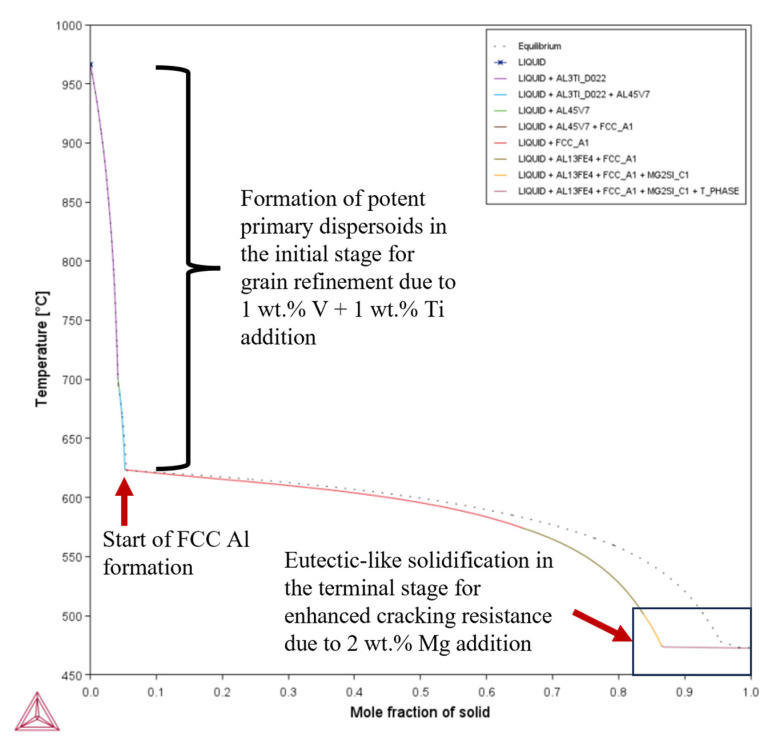
*T*–*f*_S_ curve of Composition 7 (Al 7075 + 1 wt.% V + 1 wt.% Ti. + 2 wt.% Mg alloy).

Figure 1, Figure A1, Figure A2, Figure A3, Figure A4, Figure A5 and Figure A6 present the *T*–$f_{S}$ curves of the compositions of interest from the liquidus temperature (*T*_Liquidus_) at $f_{S}$ = 0 to the end of the solidification process, where $f_{S}$ = 1, and the legends display the respective phases that form from *T*_Liquidus_ to the end of the Scheil simulation. Figure 2 compares the *T*–$f_{S}$ curves of the compositions of interest from $f_{S}$ = 0 to $f_{S}$ = 1, and Figure 3 and Figure 4 compare the *T*–$f_{S}$ and *T*–$f_{S}^{0.5}$ curves of the compositions of interest at the initial and final stages of solidification, respectively. Additionally, Table 3 and Table A1 present the various solidification indices obtained from these *T*–$f_{S}$ curves.

Two portions of the *T*–$f_{S}$ curves are important: the initial solidification stage ($f_{S}$ < 0.1) and the final solidification stage, which extends up to the end of solidification when $f_{S}$ = 1. Figure 1, Figure A1, Figure A2, Figure A3, Figure A4, Figure A5 and Figure A6 show that the initial and final stages of solidification change with the addition of the alloying elements, respectively. For Composition 1, the FCC-Al phase began forming immediately below *T*_Liquidus_ = 659.58 °C at *T* = 634.97 °C and $f_{S}$ = ~0.00333, with a minute initial freezing range of 24.61 °C (Figure 3 and Table 3), indicating that there were few dispersoids (due to the default alloying elements) to promote HN of Al grains. HN is vital for enabling printability–heterogeneity synergy. However, for Compositions 2–7, due to the introduced grain-refining elements, the primary dispersoids solidified first from *T*_Liquidus_ to the start of FCC-Al formation, with an increased initial freezing range (Figure 3 and Table 3), thereby providing potent primary particles upon which the HN of the Al grains will occur. The HN of Al grains upon the dispersoids probably formed Al_3_V and/or Al_3_Ti phases, thereby refining the grains. For Compositions 2–4, which have 1 wt.% V addition, the FCC-Al phase began forming at $f_{S}$ = ~0.0335–0.0338, whereas for Compositions 5–7, which have 1 wt.% V and 1 wt.% Ti additions, the FCC-Al phase began forming at $f_{S}$ = ~0.0515–0.0528 (Figure 3). This discrepancy is probably because the combination of 1 wt.% V and 1 wt.% Ti has a more pronounced grain-refining effect than 1 wt.% V. These behavioral characteristics that were observed with the addition of 1 wt.% V and 1 wt.% Ti align with several LPBF experimental observations. Tan et al. [33] and Li et al. [47] reported that adding 1 wt.% Ti and 4 wt.% Ti–6Al–4V alloy powders refined Al-7075 grains and eliminated cracks through the in situ formation of Al_3_Ti and Al_3_(Ti_x_,V_1−x_) phases, respectively. Similarly, Yu et al. [77], Martin et al. [36], and Li et al. [58] showed that the addition of 1 wt.% Zr, hydrogen-stabilized Zr particles, and 1 wt.% Zr produced Al_3_Zr particles that promoted equiaxed grain growth and crack resistance, respectively. In the study by Choe et al. [79], ZrH_2_ particles were introduced into Al-7075 powder, decomposing during LPBF to form Al_3_Zr in situ, which acted as potent nucleants that stimulated CET, increased the equiaxed-grain fraction, and suppressed crack initiation. Zhang et al. [76] also found that 2 wt.% Er additions reduced crack density via Al_3_Er-induced grain refinement but increased porosity due to deteriorated melt-pool fluidity. Yang et al. [80] achieved dense, crack-free Al-7075 by incorporating 5 wt.% Zr_50.7_–Cu_28_–Al_12.3_–Ni_9_ metallic-glass powders that formed Al_3_Zr and nanosized amorphous particles, resulting in strong grain refinement and improved mechanical properties. Overall, these findings corroborate the present simulation results, confirming that alloy compositions promoting early dispersoid formation—such as Al_3_V, Al_3_Ti, or Al_3_Zr— in the early solidification stage enhance HN and grain refinement in LPBF-processed Al 7xxx alloys. The CALPHAD-predicted increase in initial freezing range and steeper T–$f_{S}$ slope at $f_{S}$ < 0.1 for Compositions 2–7 is therefore consistent with experimentally verified CET promotion mechanisms.

As explained by Mishra et al. [35], a typical T–$f_{S}$ curve of an alloy with good printability should give an appearance of the letter “L,” where the nonhorizontal and horizontal lines represent the formations of primary dispersoids at the initial stage and eutectics at the terminal stage of solidification, respectively. A steeper nonhorizontal line (indicating a high formation rate of a constitutionally undercooled zone) and a longer nonhorizontal line (indicating a wider initial freezing range) are beneficial for improving printability, as evident in Compositions 2–7, which have been explicated above. Also, a longer horizontal line aided by the formation of a terminal eutectic (as indicated by the solidification path) would mean a lower HCS of the terminal stage.

Concerning the final solidification stage, relative to other alloys, Composition 1 exhibited high HSI and *T*_CR_ values of 279.29 °C and 9.47 °C, respectively, and a wider temperature range at the end of solidification (Table 3). This trend occurred because the end of the solidification process was characterized by the solidification of different phases at different temperatures (Table A2), such as the eutectic T phase (Al_2_Mg_3_Zn_3_) at *T* = 471.08 °C and $f_{S}$ = ~0.9180, Al_7_Cu_2_Fe at *T* = 470.59 °C and $f_{S}$ = 0.9184, S phase (Al_2_CuMg) at *T* = 470.57 °C and $f_{S}$ = 0.9192, and V phase ((Al, Zn)_5_(Zn, Cu)_6_(Mg)_2_)) at *T* = 462.21 °C and $f_{S}$ = 0.9850, after the solidification of the Al¬–Mg_2_Si eutectic at *T* = 503.07 °C and $f_{S}$ = 0.8977, which steepened the slope of the curve during the final solidification stage (Figure 4). Even with the addition of alloying elements, Compositions 2 and 5 displayed similar trends (Table A2). Consequently, Compositions 2 and 5 were characterized by HSI values of 263.25 °C and 253.80 °C and *T*_CR_ values of 9.40 °C and 2.71 °C, respectively. These improvements compared to Composition 1 are attributed to the alloying elements of V in Composition 2 and V and Ti in Composition 5. However, as the Mg content increased in Compositions 3, 4, 6, and 7, only the T phase (Al_2_Mg_3_Zn_3_) solidified up to $f_{S}$ = 1 after the solidification of the Al¬–Mg_2_Si eutectic, which favored cracking resistance. Consequently, these alloys exhibited relatively low HSI and *T*_CR_ (Table 3). Thus, it can be concluded that the addition of Mg favored the solidification of the desired T phase (Al_2_Mg_3_Zn_3_) eutectic as early as possible up to the end of solidification. Besides, Table A2 indicates that as the Mg content increased, the temperatures for the formation of the terminal T phase (Al_2_Mg_3_Zn_3_) eutectic increased. However, herein, the maximum Mg addition was restricted to 2 wt.% to prevent significant compositional variation. These results agree with earlier LPBF findings where Si additions improved terminal solidification and crack resistance. Montero Sistiaga et al. [75] and Otani et al. [56,57] reported that adding 4–5 wt.% Si refined grains, enhanced melt fluidity, and promoted Al–Si eutectic formation, which reduced microcracks and increased density. Likewise, Aversa et al. [78] mixed Al–Si10–Mg and Al 7075 (Al–Zn–Mg–Cu) alloy powders to obtain a new composition of 50% Al 7075 (Al–Si-Zn–Mg–Cu) alloy and observed that Si enrichment narrowed the solidification range and minimized thermal stresses. Meanwhile, Li et al. [58] added 2.9 wt.% Si and 1 wt.% Zr to Al–Zn–Mg–Cu and showed that combining Si and Zr generated Si-rich eutectics and Al_3_Zr phases that filled intergranular cracks and refined grains. In contrast to Li et al. [47] who added 4 wt.% Ti–6Al–4V alloy to Al 7075 alloy and eliminated hot cracking through grain refinement by the formation of Al_3_(Ti_x_,V_1−x_) nucleants, our results achieve similar refinement and crack resistance at lower additive levels (1 wt.%V and 1 wt.% Ti) and further enhances terminal eutectic feeding through Mg enrichment (1–2 wt.%), indicating a more compositionally efficient strategy for LPBF processability. These observations support our results, where increased Mg promoted the formation of a single terminal T-phase (Al_2_Mg_3_Zn_3_) eutectic, lowered the HSI and *T*_CR_ values, and improved cracking resistance during the final solidification stage.

Table 3 presents the CSC values of the compositions of interest. The CSC values decreased with the addition of the alloying elements, with the increase in the Mg content having the most effect in decreasing the solidification time during which the alloy is vulnerable to cracking. Therefore, the alloying elements introduced to the Al 7075 alloy are effective in enhancing the crack resistance of the alloy, as revealed by several solidification indices.

### 3.2. SEM Analysis

**Figure 5:**
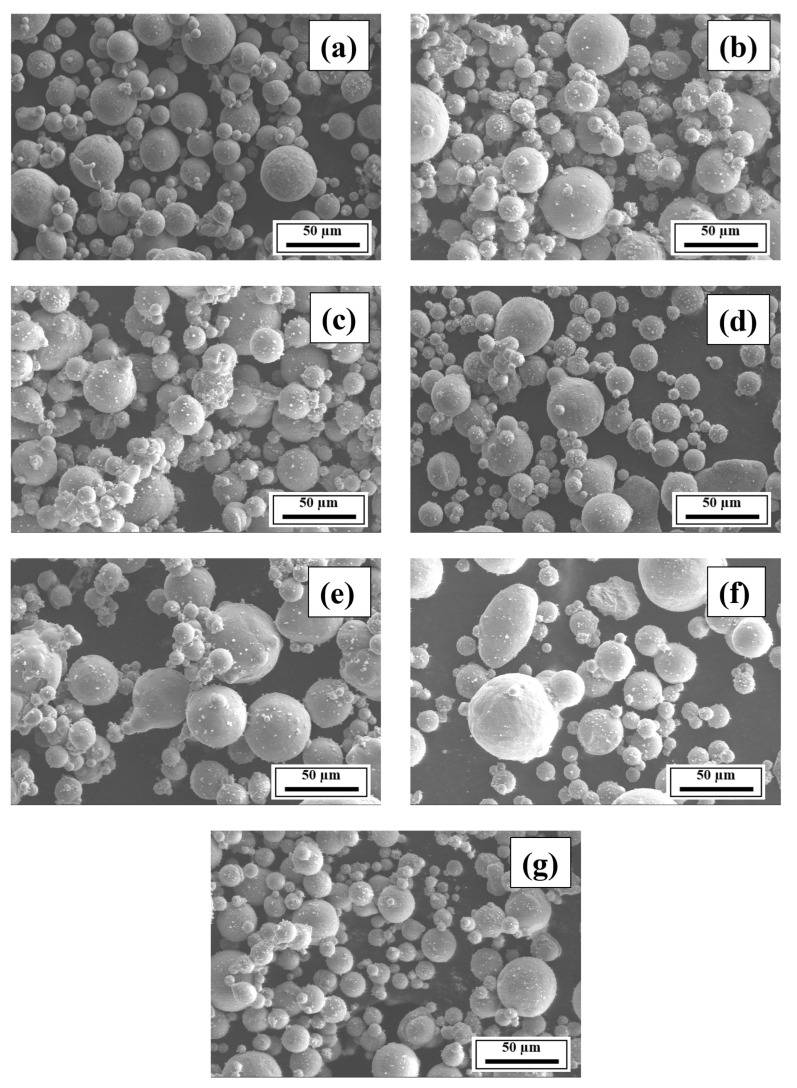
Morphology of powder samples of Compositions (**a**) 1, (**b**) 2, (**c**) 3, (**d**) 4, (**e**) 5, (**f**) 6, and (**g**) 7.

Figure 5 presents the morphology of the uniformly mixed powder samples of the compositions of interest. Compared to Figure 5a, Figure 5b–g reveal the uniform distribution of the Ti, V, and/or Mg particles on the Al 7075 alloy particles.

**Figure 6:**
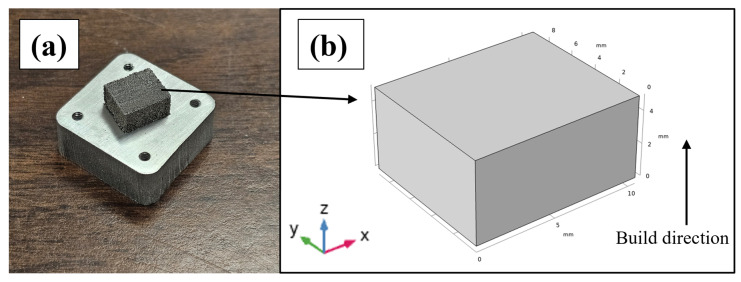
(**a**) LPBF-processed Al 7xxx alloy sample on the designed Al 7075 alloy substrate. (**b**) Dimensions of the cuboid-shaped sample.

Figure 6a shows the orientation of the LPBF-processed cuboid-shaped Al 7xxx alloy sample on the Al 7075 alloy substrate. Figure 6b reveals that the build direction (height of the cuboid) is along the *z*-axis, away from the substrate’s top horizontal surface, whereas the longest side of the cuboid is along the *x*-axis.

**Figure 7:**
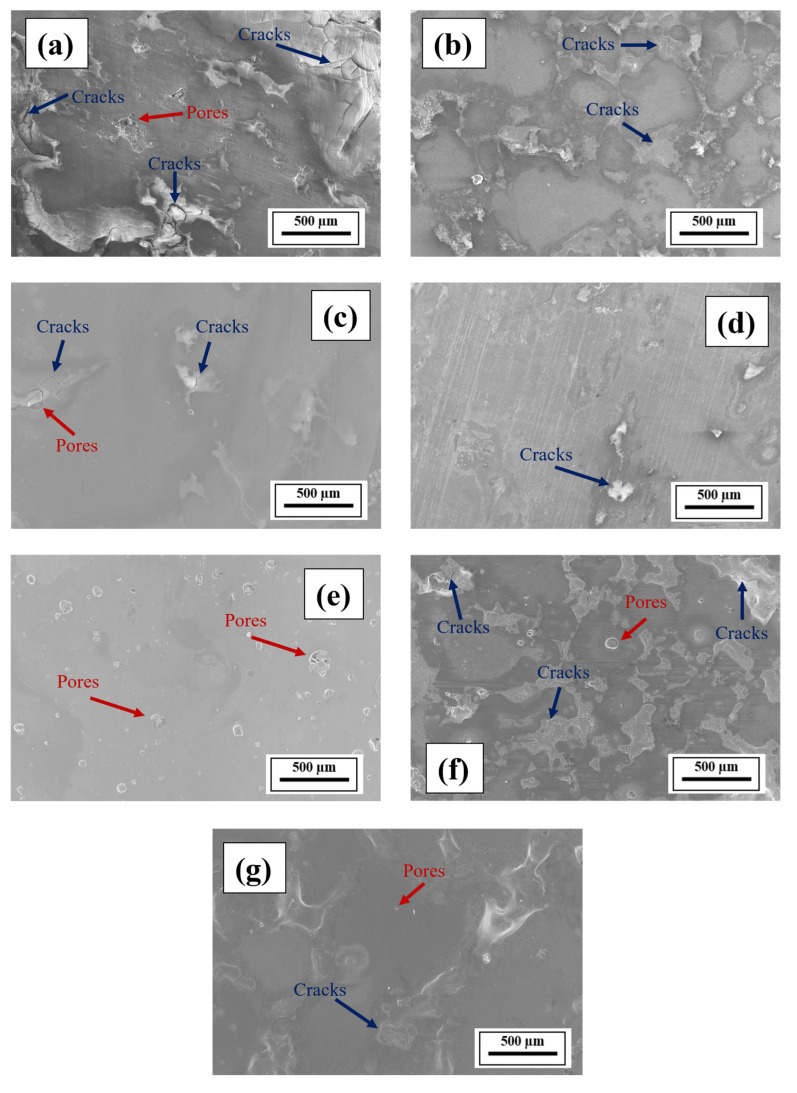
SEM images of the mirror-polished cross-sections (*y*–*z* plane) of Compositions (**a**) 1, (**b**) 2, (**c**) 3, (**d**) 4, (**e**) 5, (**f**) 6, and (**g**) 7.

For the SEM characterization, each composition was examined on the same build-direction cross-section (*y*–*z* plane) at identical magnifications and etching conditions. Multiple representative regions were inspected to ensure consistency of observed microstructural features such as grain morphology, cracks, and pores. Figure 7 shows the SEM images of the mirror-polished cross-sections (*y*–*z* plane; Figure 6b) of the LPBF-fabricated samples at a 500-µm length scale. Defects, such as micropores and microcracks, are observed. A high laser power is required to favorably wet the melt pool, which explains the residual defects observed herein [58]. Figure 7a shows significant cracks and pores in the microstructure of Composition 1 (Al 7075 alloy), probably attributed to the volumetric solidification shrinkage and thermal contraction as a result of the unavailability of sufficient liquid during the final solidification stage [32]. The microstructure comprised bright and dark regions, which are altered with the addition of alloying elements. Figure 7b–g reveal the reduction in cracks due to microstructural refinement with the addition of the alloying elements. For Composition 2 (Al 7075 + 1 wt.% V), the dark and bright regions were slightly altered, with a reduction in the crack density. For Composition 3 (Al 7075 + 1 wt.% V + 1 wt.% Mg) and Composition 4 (Al 7075 + 1 wt.% V + 2 wt.% Mg), further microstructural refinement was observed, with few cracks and pores. Surprisingly, several irregular and spherical pores were observed in Composition 5 (Al 7075 alloy + 1 wt.% Ti + 1 wt.% V). For Composition 6 (Al 7075 alloy + 1 wt.% Ti + 1 wt.% V + 1 wt.% Mg), cracks and pores were visible, which were suppressed by further increasing the Mg content. Also, while Ti and/or V caused microstructural refinement, an increase in the Mg content further suppressed the cracks due to the ES effect at the end of the solidification process, which confirmed the CALPHAD-based alloy design results. The observed cracks are parallel to the build direction, which is consistent with that observed in other LPBF-processed 2xxx, 6xxx, and 7xxx series Al alloys [36,47,81]. The reduction in crack density in Compositions 2 and 5 compared to Composition 1 can be attributed to grain refinement, which suppressed the formation and growth of crack-susceptible columnar grains [47]. In contrast, the reduction in crack density in Compositions 3, 4, 6, and 7 relative to Composition 1 can be ascribed to the combined effects of grain refinement and ES behavior at the terminal stage, which improved liquid feeding and fluidity while narrowing the final solidification range [75,78]. Although it is challenging to identify the most microstructurally refined alloy because of the residual pores and cracks resulting from the limited 168-W laser power—below the levels typically required for processing Al alloys—and the lack of process parameter optimization, the SEM results indicate that the alloying additions employed here are promising and provide a valuable baseline for future studies involving process parameter optimization.

**Figure 8:**
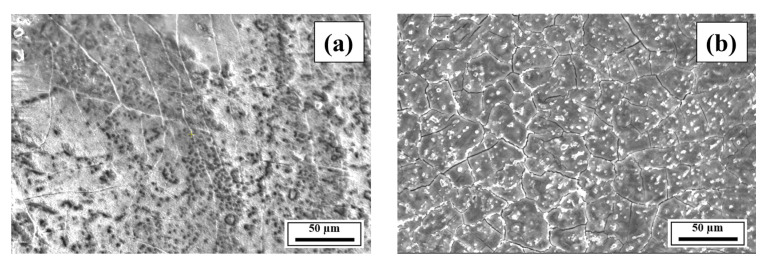

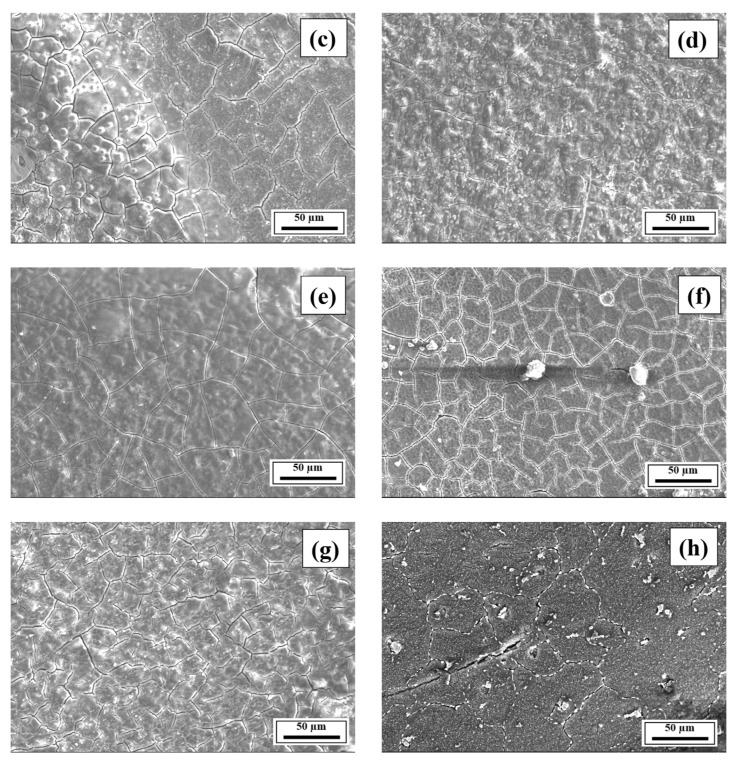
SEM images of the etched cross-sections (*y*–*z* plane) of Compositions (**a**) 1, (**b**) 2, (**c**) 3, (**d**) 4, (**e**) 5, (**f**) 6, (**g**) 7, and (**h**) side perpendicular to the extrusion direction for extruded Al 7075 alloy.

**Figure A7:**
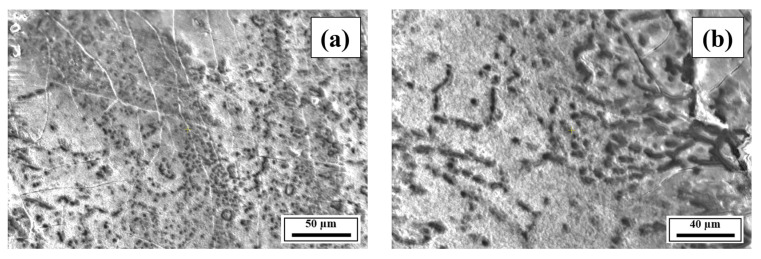

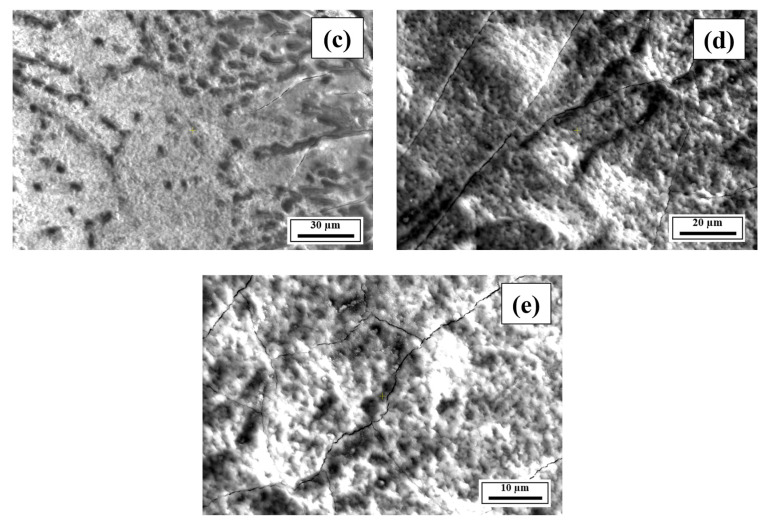
SEM images of the etched cross-section (*y*–*z* plane) of Composition 1 at (**a**) 50-, (**b**) 40-, (**c**) 30-, (**d**) 20-, and (**e**) 10-µm length scales.

**Figure A8:**
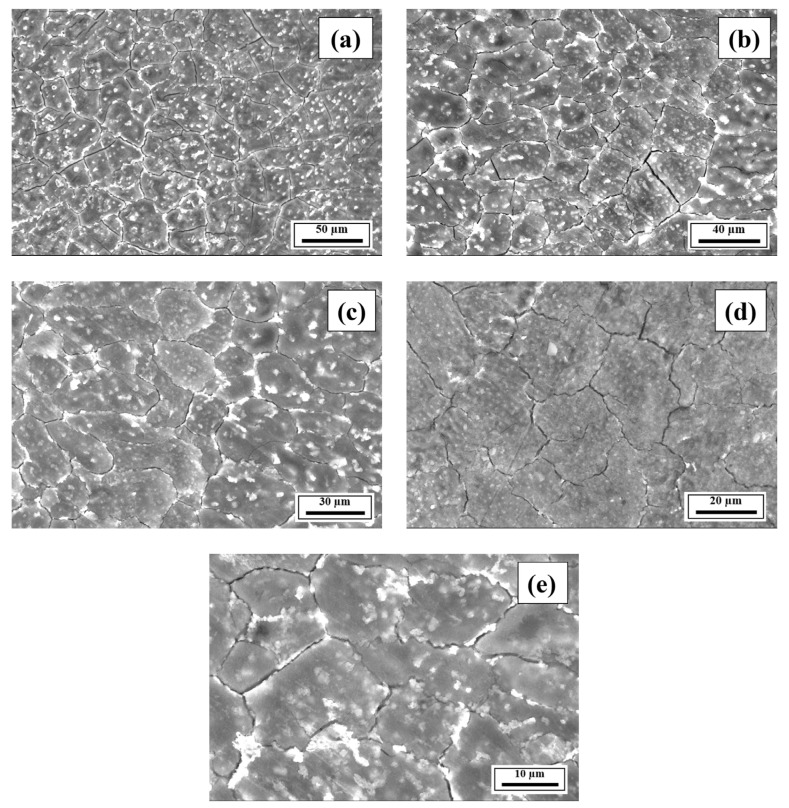
SEM images of the etched cross-section (*y*–*z* plane) of Composition 2 at (**a**) 50-, (**b**) 40-, (**c**) 30-, (**d**) 20-, and (**e**) 10-µm length scales.

**Figure A9:**
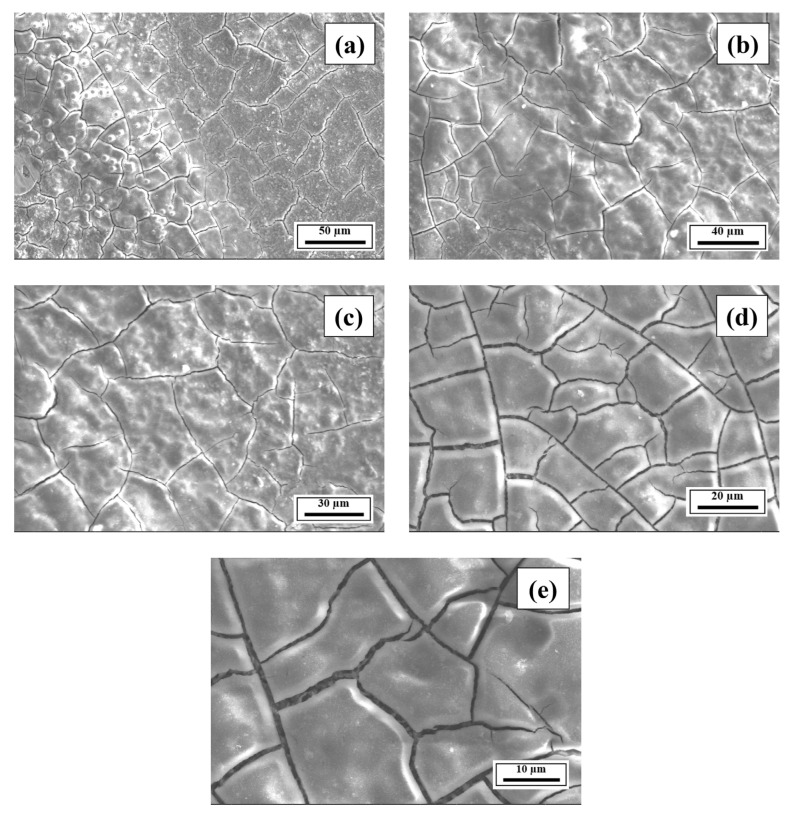
SEM images of the etched cross-section (*y*–*z* plane) of Composition 3 at (**a**) 50-, (**b**) 40-, (**c**) 30-, (**d**) 20-, and (**e**) 10-µm length scales.

**Figure A10:**
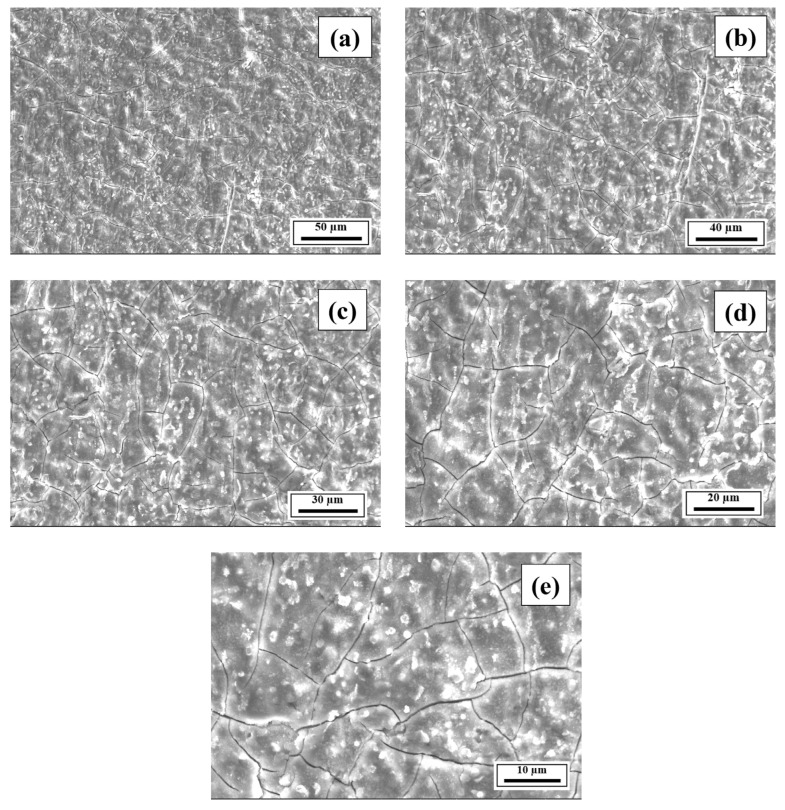
SEM images of the etched cross-section (*y*–*z* plane) of Composition 4 at (**a**) 50-, (**b**) 40-, (**c**) 30-, (**d**) 20-, and (**e**) 10-µm length scales.

**Figure A11:**
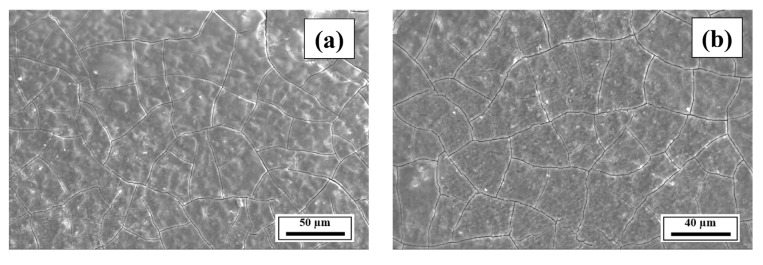

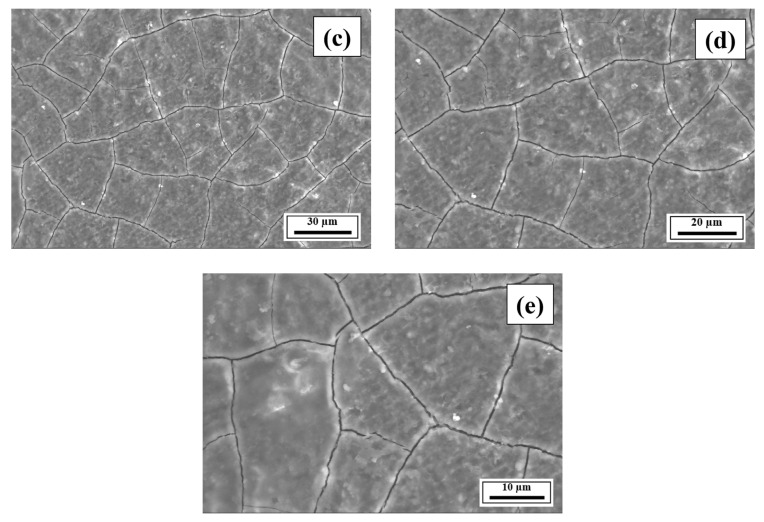
SEM images of the etched cross-section (*y*–*z* plane) of Composition 5 at (**a**) 50-, (**b**) 40-, (**c**) 30-, (**d**) 20-, and (**e**) 10-µm length scales.

**Figure A12:**
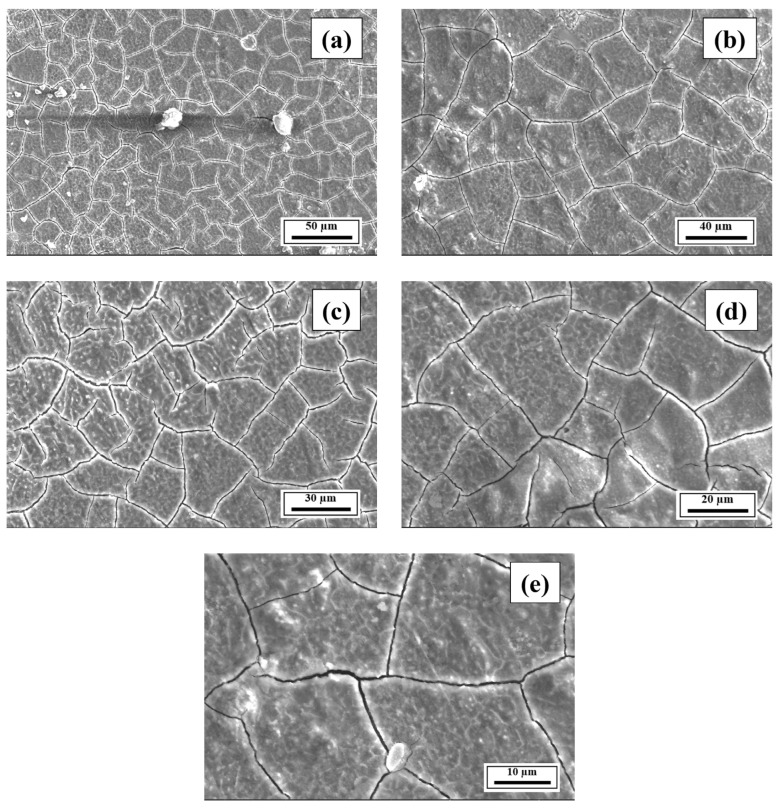
SEM images of the etched cross-section (*y*–*z* plane) of Composition 6 at (**a**) 50-, (**b**) 40-, (**c**) 30-, (**d**) 20-, and (**e**) 10-µm length scales.

**Figure A13:**
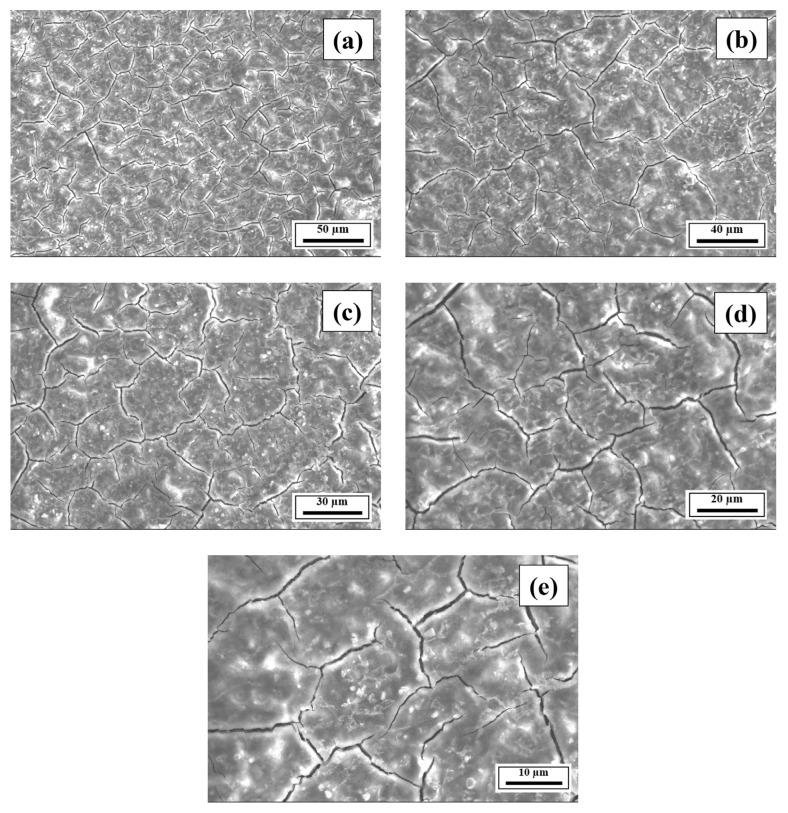
SEM images of the etched cross-section (*y*–*z* plane) of Composition 7 at (**a**) 50-, (**b**) 40-, (**c**) 30-, (**d**) 20-, and (**e**) 10-µm length scales.

**Figure A14:**
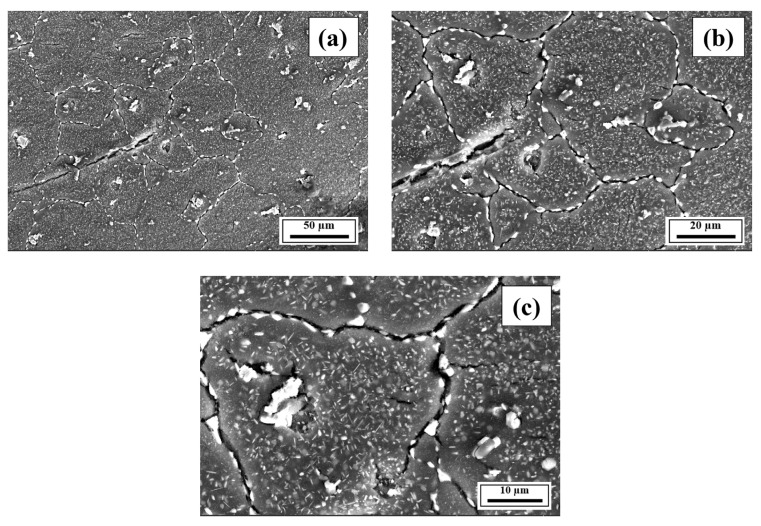
SEM images of the etched cross-section perpendicular to the extrusion direction of conventionally fabricated Al 7075 alloy at (**a**) 50-, (**b**) 20-, and (**c**) 10-µm length scales.

Figure 8 compares the SEM images of the etched cross-sections (*y*–*z* plane) of the LPBF-processed samples at a 50-µm length scale, which highlights the grain morphologies of the samples, including that of conventionally fabricated Al 7075 alloy. Compared to Composition 1, Compositions 2–7 presented fine microstructures with equiaxed grains and clearly outlined grain boundaries similar to those of conventionally fabricated Al 7075 alloy. Also, white aluminum oxide particles were dispersed on the grains and grain boundaries of all compositions. Figure A7, Figure A8, Figure A9, Figure A10, Figure A11, Figure A12, Figure A13 and Figure A14 show the SEM images of the etched cross-sections (*y*–*z* plane) of the LPBF-fabricated samples and conventionally fabricated Al 7075 alloy at varying length scales of 10–50 µm, with increments of 10 µm. For Composition 1, columnar grains were observed (Figure 8a and Figure A7) in the etched microstructure, and an avalanche of interconnected dark-colored pores formed long cracks. Decreasing the length scale (Figure A7) further revealed the numerous pores within the microstructure. This trend explains the increased material removal rate observed during metallographic preparations. Figure A8, Figure A9, Figure A10, Figure A11, Figure A12 and Figure A13 clearly show the etched microstructures of Compositions 2–7, respectively. With the addition of the alloying elements, microstructural refinements in the form of CET were observed (Figure 8, Figure A8, Figure A9, Figure A10, Figure A11, Figure A12 and Figure A13), with the grain boundaries clearly outlined, similar to the visibly outlined equiaxed grains of conventionally fabricated Al 7075 alloy (Figure A14).

**Figure A15:**
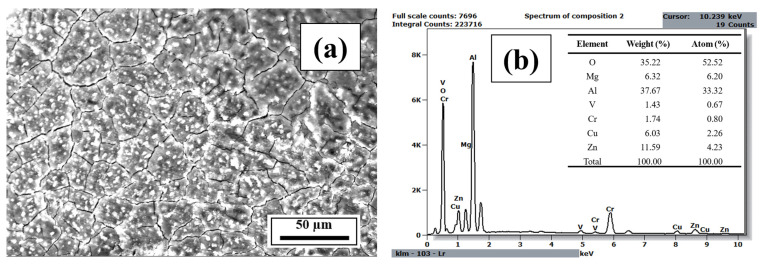
(**a**) SEM image of the etched cross-section (*y*–*z* plane) of Composition 2 (Al 7075 + 1 wt.% V alloy) and (**b**) the corresponding EDS spectrum with quantified elemental composition.

**Figure A16:**
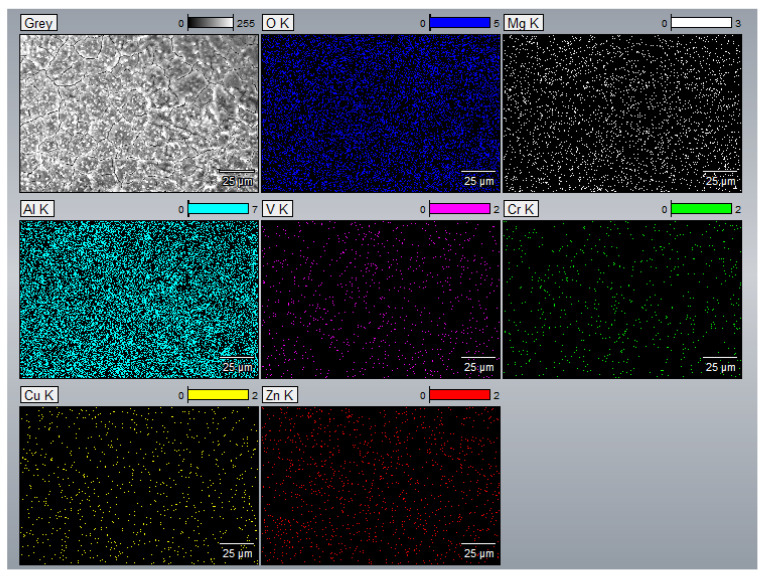
Elemental mapping of Composition 2 (Al 7075 + 1 wt.% V alloy).

**Figure A17:**
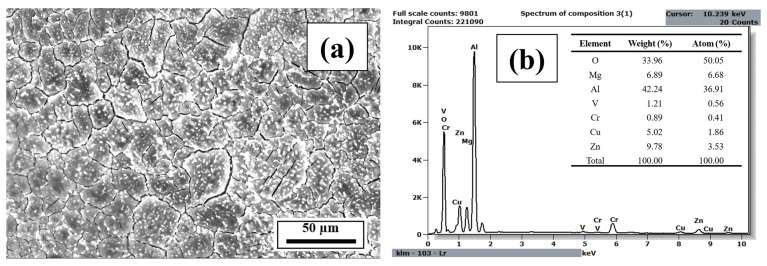
(**a**) SEM image of the etched cross-section (*y*–*z* plane) of Composition 3 (Al 7075 + 1 wt.% V + 1 wt.% Mg alloy) and (**b**) the corresponding EDS spectrum with quantified elemental composition.

**Figure A18:**
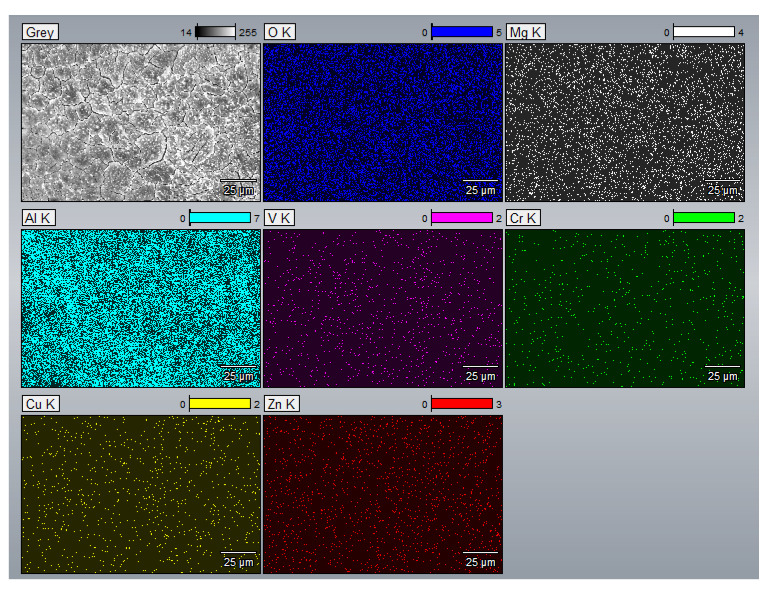
Elemental mapping of Composition 3 (Al 7075 + 1 wt.% V + 1 wt.% Mg alloy).

**Figure A19:**
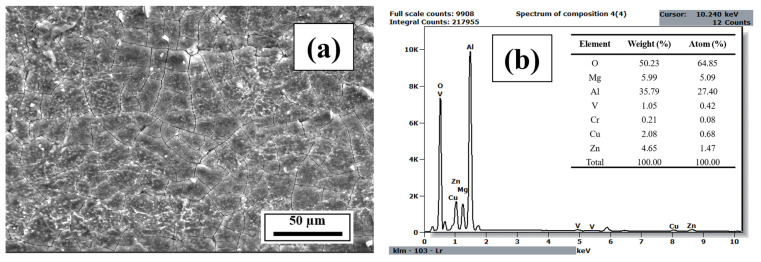
(**a**) SEM image of the etched cross-section (*y*–*z* plane) of Composition 4 (Al 7075 + 1 wt.% V + 2 wt.% Mg alloy) and (**b**) the corresponding EDS spectrum with quantified elemental composition.

**Figure A20:**
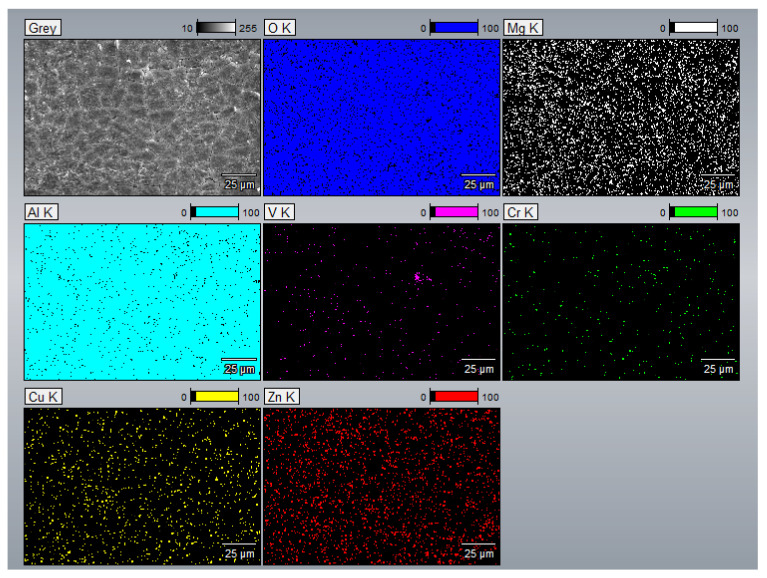
Elemental mapping of Composition 4 (Al 7075 + 1 wt.% V + 2 wt.% Mg alloy).

**Figure A21:**
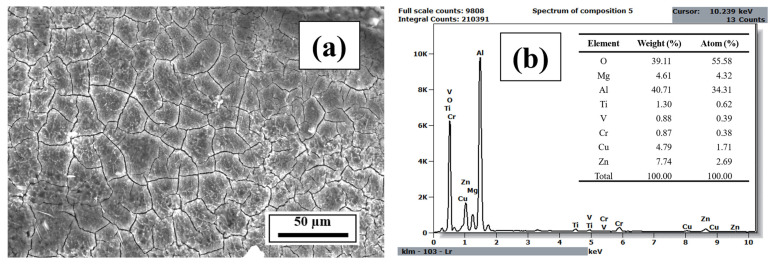
(**a**) SEM image of the etched cross-section (*y*–*z* plane) of Composition 5 (Al 7075 + 1 wt.% V + 1 wt.% Ti alloy) and (**b**) the corresponding EDS spectrum with quantified elemental composition.

**Figure A22:**
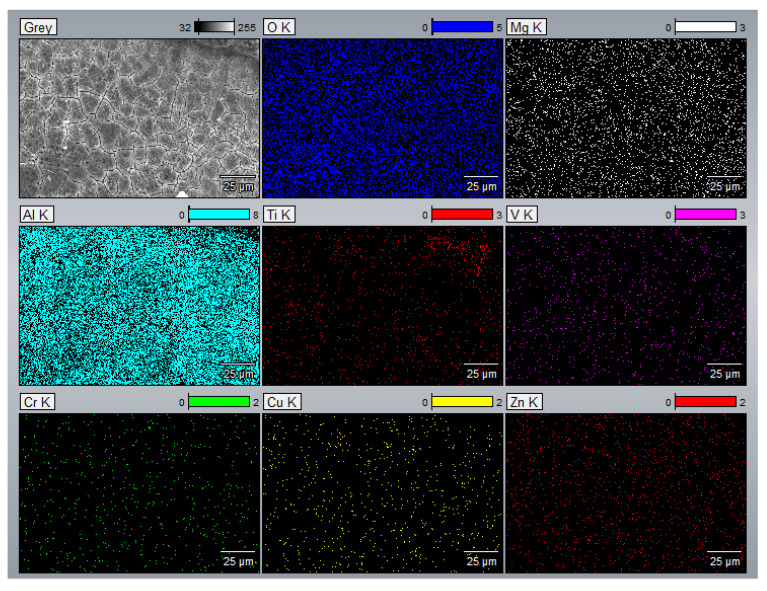
Elemental mapping of Composition 5 (Al 7075 + 1 wt.% V + 1 wt.% Ti alloy).

**Figure A23:**
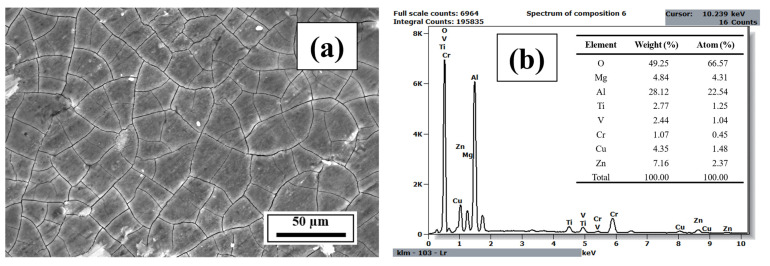
(**a**) SEM image of the etched cross-section (*y*–*z* plane) of Composition 6 (Al 7075 + 1 wt.% V + 1 wt.% Ti + 1 wt.% Mg alloy) and (**b**) the corresponding EDS spectrum with quantified elemental composition.

**Figure A24:**
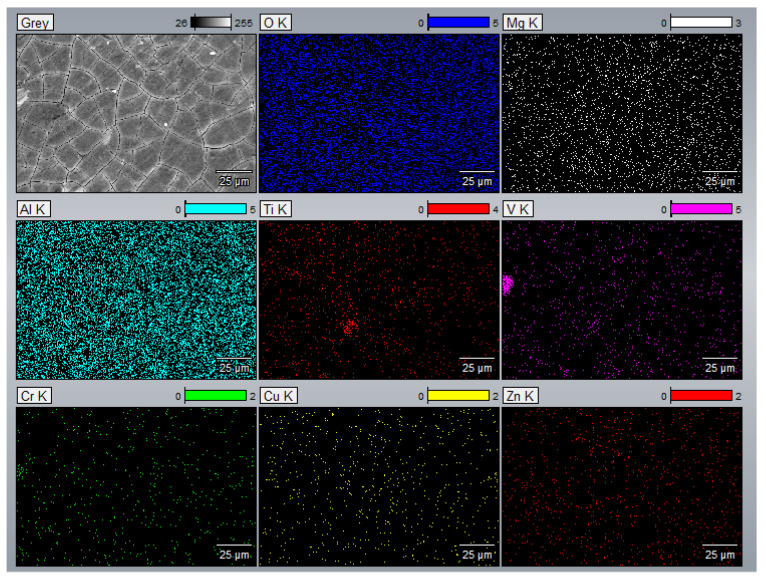
Elemental mapping of Composition 6 (Al 7075 + 1 wt.% V + 1 wt.% Ti + 1 wt.% Mg alloy).

**Figure A25:**
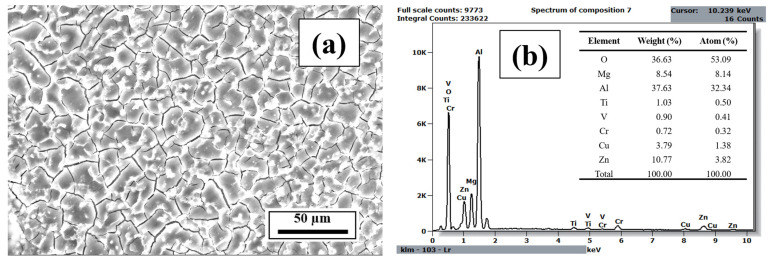
(**a**) SEM image of the etched cross-section (*y*–*z* plane) of Composition 7 (Al 7075 + 1 wt.% V + 1 wt.% Ti + 2 wt.% Mg alloy) and (**b**) the corresponding EDS spectrum with quantified elemental composition.

**Figure A26:**
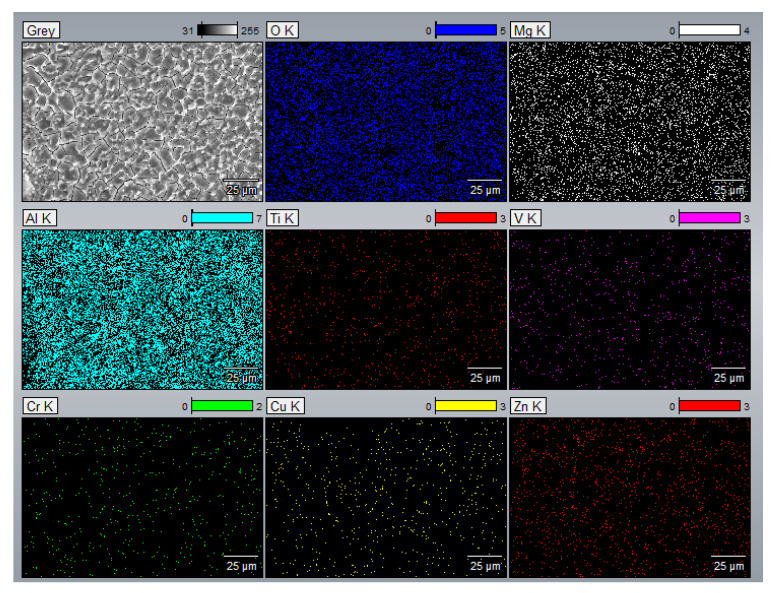
Elemental mapping of Composition 7 (Al 7075 + 1 wt.% V + 1 wt.% Ti + 2 wt.% Mg alloy).

The addition of V in Compositions 2 and 5 caused the formation of Al_3_V, whereas the addition of V and Ti in Compositions 3, 4, 6, and 7 led to the formation of Al_3_V and Al_3_Ti. These L1_2_ primary trialuminides with small sizes and high densities are uniformly distributed all around the matrices of Compositions 2–7 (Figure A15, Figure A16, Figure A17, Figure A18, Figure A19, Figure A20, Figure A21, Figure A22, Figure A23, Figure A24, Figure A25 and Figure A26). They facilitate the formation of fine, crack-resistant equiaxed grains and limit the growth of large columnar grains. They act as ideal nuclei for the primary face-centered cubic (FCC) Al phase because of their structural similarity to the FCC Al phase [35] and exert strong pinning effects on the grain boundaries, thereby limiting their movement and grain growth, leading to the grain refinement [58]. The grain refinement increases the grain boundaries per unit volume, which largely decreases the thickness of the residual liquid film at the final solidification stage, thereby decreasing the initiation and propagation of cracks [58]. Similar grain refinement effects have been reported for LPBF-processed Al 7xxx alloys due to the in situ formation of Al_3_Ti, Al_3_(Ti_x_,V_1−x_), Al_3_Zr, and Al_3_Er [33,36,47,58,76,77,79,80]. The addition of Mg in Compositions 3, 4, 6, and 7 induced the formation of Mg-rich eutectics, which decreased the crack sensitivity and improved the fluidity at the final solidification stage. A similar outcome was reported by Li et al. [58] for Al 7xxx alloys by adding 2.9 wt.% Si. It is assumed that a high laser power will favor the formation of more Mg-rich eutectics due to the relatively higher temperature generated by the increased laser power, which will reduce the number of defects. Similar eutectic effects have been reported for LPBF-processed Al 7xxx alloys due to additions of Si [56,57,58,75,78]. Thus, the grain refinement due to the addition of V and/or Ti and the eutectic formation by the addition of Mg synergistically inhibited/reduced the formation of hot cracks during the LPBF process. Li et al. [58] reported a similar synergistic effect for Al 7xxx alloys by adding 2.9 wt.% Si and 1 wt.% Zr.

### 3.3. EDS Analysis

**Figure 9:**
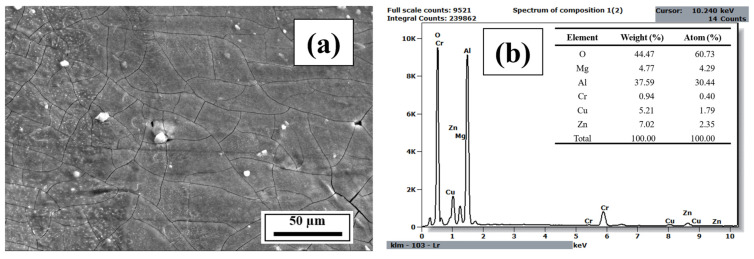
(**a**) SEM image of the etched cross-section (*y*–*z* plane) of Composition 1 (Al 7075 alloy) and (**b**) the corresponding EDS spectrum with quantified elemental composition.

**Figure 10:**
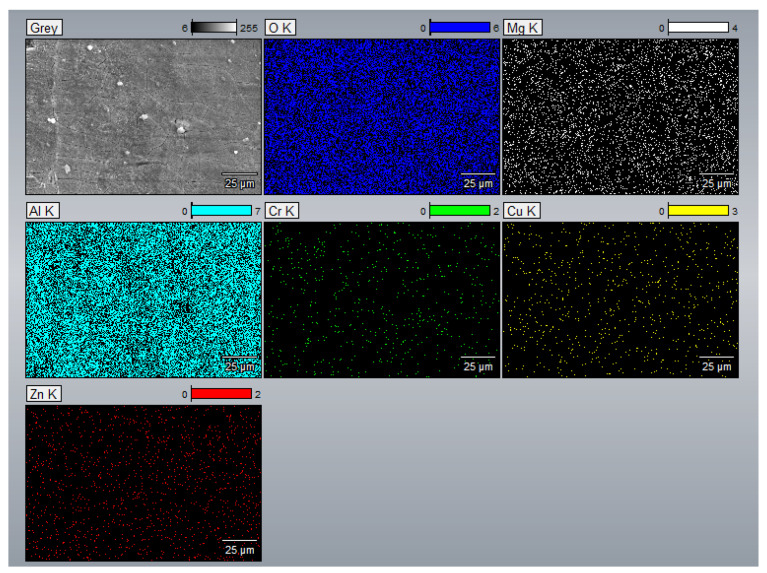
Elemental mapping of Composition 1 (Al 7075 alloy).

EDS spectra and elemental mappings were acquired from multiple representative regions per composition under identical beam and detector settings to confirm compositional uniformity. This approach minimized local variability and ensured consistent semi-quantitative analysis across all samples. Figure 9, Figure 10, Figure A15, Figure A16, Figure A17, Figure A18, Figure A19, Figure A20, Figure A21, Figure A22, Figure A23, Figure A24, Figure A25 and Figure A26 present the spectra, quantitative elemental analysis, and elemental mappings of Compositions 1–7. The SEM images used for the spectral analysis of Compositions 1–7 displayed white-colored aluminum oxide phases in the samples. This trend is probably because aluminum is easily oxidized. Also, the corresponding quantitative elemental mappings of Compositions 1–7 displayed a significant amount of oxygen in the samples. Additionally, the elemental mappings of Compositions 1–7 indicated that the alloying elements were uniformly distributed in the samples. Figure 9a also shows the columnar grains of Composition 1 as described in the SEM study, whereas Figure A15, Figure A17, Figure A19, Figure A21, Figure A23 and Figure A25 reveal the equiaxed grains of the refined microstructures due to CET.

### 3.4. X-Ray Micro-CT Analysis 

Table 4 presents the summary statistics of the X-ray micro-CT results. Each X-ray micro-CT scan encompassed the full printed specimen volume, eliminating sampling bias. Reported metrics (mean pore size, total pore volume, and pore-volume-to-sample-volume ratios) therefore represent complete volumetric measurements. To understand the results here, it would be necessary to view the as-printed samples in three categories: Category 1, which represents Al 7075 alloy (Composition 1); Category 2, which represents Category 1 + grain refiners, such as V and/or Ti, (*i.e.*, Compositions 2 and 5); Category 3, which represents Category 2 + an ES enhancer, such as Mg (*i.e.*, Compositions 3, 4, 6, and 7). The volume of pores in the as-printed samples ranged from 0 to 10^19^ nm^3^, and the mean pore volumes of the as-printed samples were in the order of 10^−5^ mm^3^. For Category 2, the mean pore volume decreased and increased for Compositions 2 and 5, respectively, compared to Composition 1. For Category 3, the mean pore volume increased significantly as the Mg content increased. Also, Table 4 presents the percentage porosity decrement of the as-printed samples compared to Composition 1, indicating improved porosity reduction in Compositions 2–7. Since the tested samples varied in volume due to metallographic processing challenges, the percentage porosity decrement with respect to Composition 1 was obtained by calculating the ratio of the volume of pores to the total volume of each sample. This result signifies that the addition of grain refiners and an ES enhancer, as adopted in this study, is satisfactory in reducing/eliminating cracks and pores in LPBF-processed Al 7xxx alloys. Despite the ~7%–28% porosity reduction observed in the as-printed Compositions 2–7 compared to Composition 1, pores were still present in Compositions 2–7 due to the process parameters adopted in this study. The samples were printed at a laser power *P*_L_ of 168 W, which is the maximum power of the custom-built SLM equipment, and a scan speed *V*_L_ of 500 mm·s^−1^. Achieving denser parts in future studies will require high laser power *P*_L_ of 300–350 W to ensure complete powder melting, addressing the challenges posed by the high reflectivity and thermal conductivity of Al alloys. Also, future research will require optimizing the LPBF parameters with substrate preheating and modification to eliminate defects. Nevertheless, the results presented here provide a critical baseline for optimizing process parameters in subsequent studies to enhance the quality of LPBF-processed Al alloy components.

### 3.5. XRD Analysis

**Figure 11:**
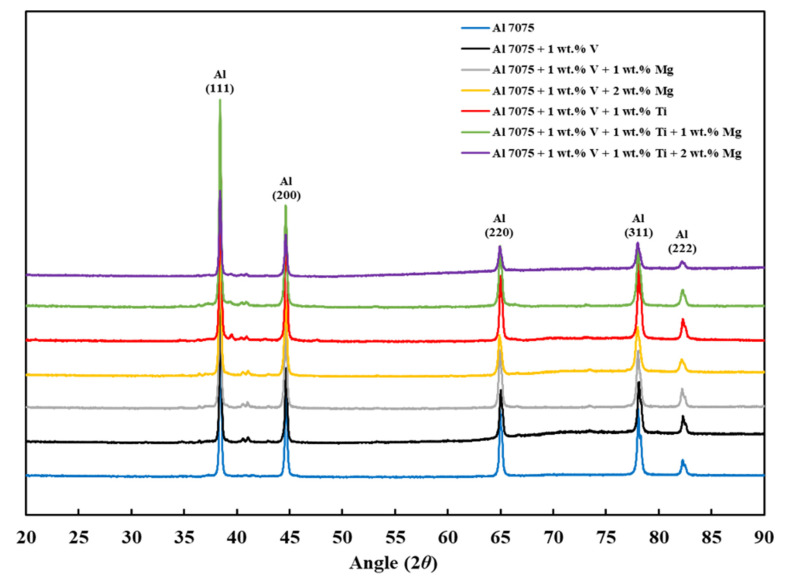
XRD spectra of the as-built samples from 20° to 90°.

**Figure 12:**
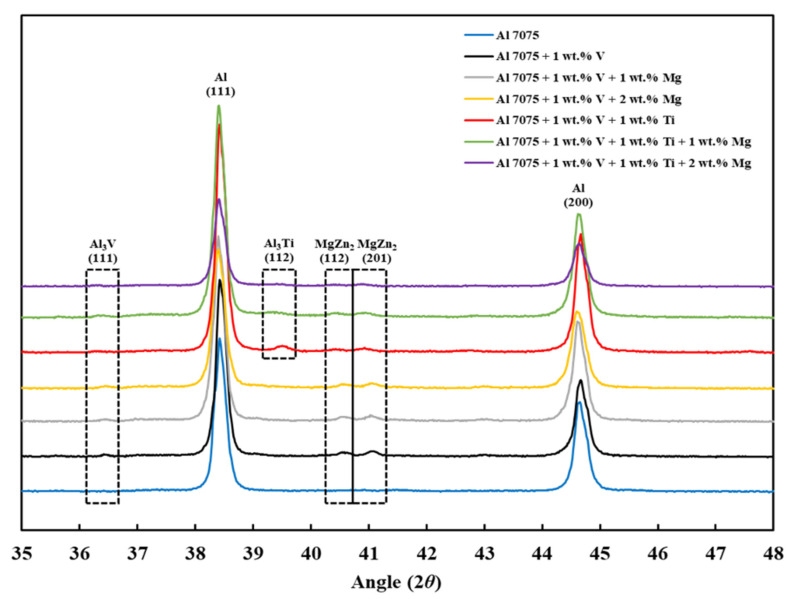
XRD spectra of the as-built samples from 35° to 48°.

**Figure 13:**
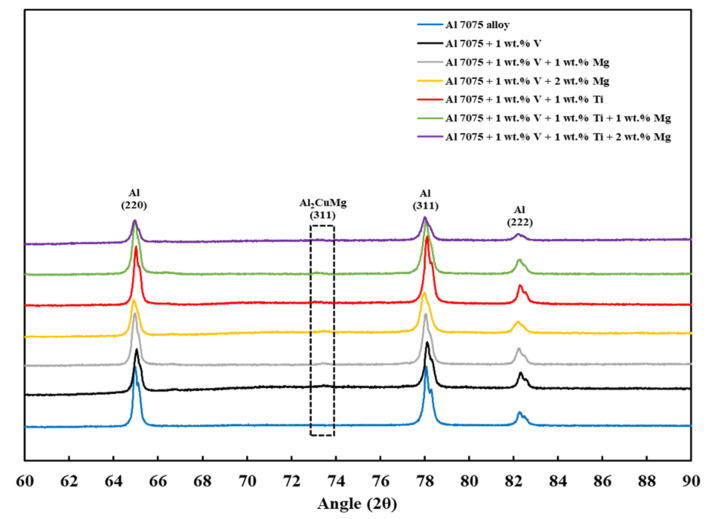
XRD spectra of the as-built samples from 60° to 90°.

Figure 11 shows the XRD *θ*/2*θ* results of the LPBF-processed samples (Compositions 1–7). Each XRD measurement was performed on the entire *x*–*y* plane of the as-built sample using identical scan parameters, ensuring reproducible phase identification and minimizing alignment or orientation-related errors. For all samples, the (111), (200), (220), (311), and (222) main crystallographic orientations of aluminum were observed, with the (111) and (200) crystallographic orientations having the highest intensities in decreasing order. Figure 12 and Figure 13 show other phase constituents of the as-printed samples, indicating that Al_2_CuMg and MgZn_2_ phases were present in all as-printed samples. The Al_3_V phase was present in Compositions 2–7, and the Al_3_Ti phase appeared in Compositions 5–7, respectively. The detected Al_3_V and Al_3_Ti phases confirmed the results of the CALPHAD-based alloy design (Section 3.1) and the microstructural refinement observed in the SEM results (Section 3.2). These well-dispersed V and Ti particles act as HN sites to induce significant grain refinement due to the small lattice misfit with the Al matrix. This trend is consistent with prior LPBF studies showing that Ti-based inoculants (e.g., Ti and Ti/TiN nanoparticles) resulted in grain refinement [38,54]. The structures of the Al_3_Ti and Al_3_V phases are FCC L1_2_, with lattice parameters of 3.967 and 3.870 Å, corresponding to −2.04% and −4.44% misfit with Al, respectively [35].

## 4. Conclusions

In this study, using an integrated computational and experimental framework, the synergy of grain refinement and ES was employed to address the hot cracking and porosity issues in LPBF-processed Al 7075 alloy due to rapid solidification without significant compositional alteration and to enable printability–heterogeneity. The main conclusions of this study can be summarized as follows:

1. By adding grain refiners (V and Ti) and an ES enhancer (Mg) to Al 7075 alloy, the compositions of interest were developed using CALPHAD and categorized into three as follows: Category 1 (Composition 1 = Al 7075), Category 2 (Composition 2 = Al 7075 + 1 wt.% V and Composition 5 = Al 7075 + 1 wt.% V + 1 wt.% Ti), and Category 3 (Composition 3 = Al 7075 + 1 wt.% V + 1 wt.% Mg, Composition 4 = Al 7075 + 1 wt.% V + 2 wt.% Mg, Composition 6 = Al 7075 + 1 wt.% V + 1 wt.% Ti + 1 wt.% Mg, and Composition 7 = Al 7075 + 1 wt.% V + 1 wt.% Ti + 2 wt.% Mg).
2. Unlike for Composition 1, the *T*–$f_{S}$ curves of Compositions 2–7 from $f_{S}$= 0 to $f_{S}$ = 0.1 revealed that the primary dispersoids (due to Vand/or Ti) solidified first from *T*_Liquidus_ to the start of FCC-Al formation, with an increased freezing range. The HN of Al grains upon the dispersoids formed Al_3_V and/or Al_3_Ti phases, thereby refining the grains. The combined effect of 1 wt.% V and 1 wt.% Ti exceeded that of 1 wt.% V.
3. Composition 1 exhibited the highest HSI and *T*_CR_ values of 279.29 °C and 9.47 °C, respectively, at the final solidification stage. Category 2 improved in HSI (~5.8% and 9.1% decrements for Compositions 2 and 5, respectively) and *T*_CR_ (~0.74% and 71.37% decrements for Compositions 2 and 5, respectively) compared to Category 1. As the Mg content increased in Category 3, only the T phase (Al_2_Mg_3_Zn_3_) eutectic solidified up to $f_{S}$ = 1 after the solidification of the Al¬–Mg_2_Si eutectic, which favored cracking resistance. Consequently, Category 3 exhibited relatively low HSI (~86.2%, 94.6%, 92.7%, and 94.7% decrements for Compositions 3, 4, 6, and 7, respectively) and *T*_CR_ (~94.5%, 95.8%, 94.8%, and 96% decrements for Compositions 3, 4, 6, and 7, respectively) values compared to Category 1. Thus, the increase in Mg content favored the solidification of the desired T phase (Al_2_Mg_3_Zn_3_) eutectic as early as possible up to the end of solidification.
4. Composition 1 exhibited the highest CSC value of 0.419. Category 2 improved in CSC (~15.8% and 24.8% decrements for Compositions 2 and 5, respectively) compared to Category 1. As the Mg content increased in Category 3, further improvements in CSC were observed (~58.9%, 60.1%, 62.1%, and 62.8% decrements for Compositions 3, 4, 6, and 7, respectively) compared to Category 1. Thus, the addition of Mg significantly reduced the time during which the sample is vulnerable to cracking.
5. The SEM results revealed the crack reduction in the as-printed Compositions 2–7 compared to Composition 1 due to microstructural refinement and liquid availability during the final solidification stages. Also, while Ti and/or V caused microstructural refinement, an increase in the Mg content in Category 3 further suppressed the cracks due to the ES effect at the end of the solidification process, which corroborated the CALPHAD-based alloy design results using solidification indices. However, residual cracks and/or pores were observed in all the samples due to the lack of process parameter optimization.
6. The SEM results of the etched cross-sections (*y*–*z* plane) of the LPBF-fabricated samples revealed that columnar grains and an avalanche of interconnected dark-colored pores that formed long cracks were present in Composition 1. For Compositions 2–7, microstructural refinements in the form of CET were observed, with the grain boundaries clearly outlined, similar to that of conventionally fabricated Al 7075 alloy.
7. The EDS results confirmed that the alloying elements were uniformly distributed in the as-printed samples and that aluminum oxide was present in all as-printed samples.
8. The X-ray micro-CT results revealed that in terms of percentage porosity decrement of the as-printed samples compared to Composition 1, Compositions 2–7 exhibited ~7–28% improvement in porosity reduction. However, pores were still present in Compositions 2–7 due to the adopted 168-W laser power. These results will serve as a critical baseline for improved studies using high laser power suitable for Al alloys.
9. Finally, the XRD results revealed that the Al_3_V phase was observed in Compositions 2–7, and the Al_3_Ti phase was observed in Compositions 5–7, confirming the results of the CALPHAD-based alloy design and SEM results.
10. Future work will require optimizing the LPBF parameters with substrate preheating and modification to eliminate defects. In parallel, mechanical-property characterization and precipitation-hardening heat treatment optimization should be conducted to establish process–structure–property links.

## Figures and Tables

**Table 1:** Compositions of interest.

Composition	Al 7075 Alloy (wt.%)	V (wt.%)	Mg (wt.%)	Ti (wt.%)
1	100%	-	-	-
2	Balance	1	-	-
3	Balance	1	1	-
4	Balance	1	2	-
5	Balance	1	-	1
6	Balance	1	1	1
7	Balance	1	2	1

**Table 2:** Composition of spherical Al 7075 alloy.

Elements	Al	Ti	Zn	Cr	Mg	Mn	Cu	Fe	Si
Composition (wt.%)	Balance	0.01	5.35	0.19	2.62	0.01	1.61	0.07	0.05

**Table 3:** Summary of solidification indices obtained using classic Scheil simulation of CALPHAD.

Composition	CSC	*T*_Liquidus_ (°C)	T (Start of FCC Al Formation; °C)	Initial Freezing Range (°C)	T $(f_{S}$ = 0.95; °C)	T $(f_{S}$ = 1; °C)	TCR (°C)	HSI (°C)
1	0.419	659.58	$634.97\text{ @ }f_{S}$ = ~0.00333	24.61	469.11	459.64	9.47	279.29
2	0.353	837.54	$633.55\text{ @ }f_{S}$ = ~0.0335	203.99	469.05	459.65	9.40	263.25
3	0.172	843.73	$627.99\text{ @ }f_{S}$ = ~0.0335	215.74	472.57	472.05	0.52	38.55
4	0.167	849.95	$622.37\text{ @ }f_{S}$ = ~0.0338	227.58	472.95	472.56	0.39	15.09
5	0.315	952.87	$635.46\text{ @ }f_{S}$ = ~0.0515	317.41	469.01	466.30	2.71	253.80
6	0.159	959.56	$629.52\text{ @ }f_{S}$ = ~0.0519	330.04	472.58	472.09	0.49	20.26
7	0.156	966.27	$623.51\text{ @ }f_{S}$ = ~0.0528	342.76	472.90	472.52	0.38	14.92

**Table 4:** Summary statistics of X-ray micro-CT results.

Composition	Mean Pore Size (10^−5^ mm^3^)	Volume of Sample (mm^3^)	Total Volume of Pores in the Sample (mm^3^)	Ratio of Pore Volume to Sample Volume	Porosity Decrement Compared to Composition 1 (%)
1	1.48	60.94 ± 3.81	6.86	0.113 ± 0.0071	-
2	0.15	330.71 ± 4.17	27.18	0.0822 ± 0.0010	27.11
3	0.11	332.11 ± 3.29	26.98	0.0813 ± 0.0008	27.84
4	4.16	183.80 ± 3.11	13.53	0.0736 ± 0.0012	34.62
5	7.39	383.31 ± 5.34	38.03	0.0992 ± 0.0014	11.88
6	0.14	375.25 ± 5.19	39.66	0.1057 ± 0.0015	6.14
7	4.63	143.07 ± 4.28	14.85	0.1038 ± 0.0031	7.81

## Data Availability

The original contributions presented in this study are included in the article. Further inquiries can be directed to the corresponding author.

## Abbreviations

The following abbreviations are used in this manuscript:

AM	Additive manufacturing
CALPHAD	Calculation of Phase Diagrams
CET	Columnar-to-equiaxed transition
CSC	Cracking susceptibility coefficient
EDX	Energy-dispersive X-ray spectroscopy
ES	Eutectic solidification
FCC	Face-centered cubic
HCS	Hot cracking susceptibility
HN	Heterogenous nucleation
HSI	Hot susceptibility index
LPBF	Laser powder bed fusion
Micro-CT	Micro-computed tomography
SEM	Scanning electron microscopy
SLM	Selective laser melting
VED	Volumetric energy density
XRD	X-ray diffraction

## Appendix A

### Appendix A.1. CALPHAD-Based Alloy Design Results

**Table A1:** Solidus and liquidus temperatures and freezing ranges of the compositions of interest.

Composition	*T*_Solidus_ (°C)	*T*_Liquidus_ (°C)	Freezing Range (°C)
1	494.06	659.58	165.52
2	483.73	837.54	353.82
3	478.09	843.73	365.63
4	473.03	849.95	376.91
5	483.21	952.87	469.68
6	477.74	959.56	481.82
7	472.85	966.27	493.43

**Table A2:** Phases present at the final stage of solidification.

Composition	Al–¬Mg_2_Si Eutectic	T Phase (Al_2_Mg_3_Zn_3_) eutectic	Al_7_Cu_2_Fe	S Phase (Al_2_CuMg)	V Phase ((Al, Zn)_5_(Zn, Cu)_6_(Mg)_2_))
1	*T* = 503.07 °C *f*_S_ = 0.8977	*T* = 471.08 °C *f*_S_ = 0.9180	*T* = 470.59 °C *f*_S_ = 0.9184	*T* = 470.57 °C *f*_S_ = 0.9192	*T* = 462.21 °C *f*_S_ = 0.9850
2	*T* = 503.65 °C *f*_S_ = 0.8955	*T* = 470.86 °C *f*_S_ = 0.9166	*T* = 470.54 °C *f*_S_ = 0.9196	*T* = 470.51 °C *f*_S_ = 0.9204	*T* = 462.03 °C *f*_S_ = 0.9850
3	*T* = 503.61 °C *f*_S_ = 0.8674	*T* = 473.32 °C *f*_S_ = 0.8924	N/A	N/A	N/A
4	*T* = 502.51 °C *f*_S_ = 0.8387	*T* = 474.28 °C *f*_S_ = 0.8674	N/A	N/A	N/A
5	*T* = 502.16 °C *f*_S_ = 0.8951	*T* = 471.12 °C *f*_S_ = 0.9152	*T* = 470.71 °C *f*_S_ = 0.9158	*T* = 469.94 °C *f*_S_ = 0.9327	*T* = 461.91 °C *f*_S_ = 0.9851
6	*T* = 502.59 °C *f*_S_ = 0.8664	*T* = 473.56 °C *f*_S_ = 0.8906	N/A	N/A	N/A
7	*T* = 501.55 °C *f*_S_ = 0.8373	*T* = 474.52 °C *f*_S_ = 0.8651	N/A	N/A	N/A
